# Alendronate as Bioactive Coating on Titanium Surfaces: An Investigation of CaP–Alendronate Interactions

**DOI:** 10.3390/ma17112703

**Published:** 2024-06-03

**Authors:** Ines Despotović, Željka Petrović, Jozefina Katić, Dajana Mikić

**Affiliations:** 1 Division of Physical Chemistry, Ruđer Bošković Institute, Bijenička Cesta 54, 10002 Zagreb, Croatia; 2 Division of Materials Chemistry, Ruđer Bošković Institute, Bijenička Cesta 54, 10002 Zagreb, Croatia; 3 Department of Electrochemistry, Faculty of Chemical Engineering and Technology, University of Zagreb, Marulićev Trg 19, 10000 Zagreb, Croatia; jkatic@fkit.unizg.hr (J.K.); dmikic@fkit.unizg.hr (D.M.)

**Keywords:** titanium dental implant, alendronate coating, calcium phosphate CaP, bioactivity, DFT quantum chemical calculations

## Abstract

The surface modification of dental implants plays an important role in establishing a successful interaction of the implant with the surrounding tissue, as the bioactivity and osseointegration properties are strongly dependent on the physicochemical properties of the implant surface. A surface coating with bioactive molecules that stimulate the formation of a mineral calcium phosphate (CaP) layer has a positive effect on the bone bonding process, as biomineralization is crucial for improving the osseointegration process and rapid bone ingrowth. In this work, the spontaneous deposition of calcium phosphate on the titanium surface covered with chemically stable and covalently bound alendronate molecules was investigated using an integrated experimental and theoretical approach. The initial nucleation of CaP was investigated using quantum chemical calculations at the density functional theory (DFT) level. Negative Gibbs free energies show a spontaneous nucleation of CaP on the biomolecule-covered titanium oxide surface. The deposition of calcium and phosphate ions on the alendronate-modified titanium oxide surface is governed by Ca^2+^–phosphonate (-PO_3_H) interactions and supported by hydrogen bonding between the phosphate group of CaP and the amino group of the alendronate molecule. The morphological and structural properties of CaP deposit were investigated using scanning electron microscopy, energy dispersive X-ray spectroscopy, X-ray diffraction and attenuated total reflectance Fourier transform infrared spectroscopy. This integrated experimental–theoretical study highlights the spontaneous formation of CaP on the alendronate-coated titanium surface, confirming the bioactivity ability of the alendronate coating. The results provide valuable guidance for the promising forthcoming advancements in the development of biomaterials and surface modification of dental implants.

## 1. Introduction

Titanium-based materials are the most commonly used for the manufacture of medical implants [1,2,3,4,5], mainly due to their suitable mechanical properties [6]. In addition, titanium and most of its alloys have shown excellent tissue compatibility [7]. However, these materials have been shown to be highly bioinert due to poor contact with the living body upon implantation [8,9]. Since the effectiveness of the osseointegration process with the surrounding tissue depends on the interactions that occur at the outermost surface layer with few atomic distances, the titanium surface has been specifically modified to improve integration with the surrounding tissue and minimize the risks associated with the release of metal ions into the body. Among a number of established approaches to surface modification (functionalization) [10], the formation of a biocompatible and bioactive layer that irreversibly adheres to the surface has attracted particular attention [11,12,13,14].

Organophosphorus compounds such as phosphonates have proven to be effective agents for the functionalization of titanium surfaces by forming homogeneous self-assembled monolayers. In particular, alendronate, a nitrogen-containing bisphosphonate, exhibits good coating properties on titanium surfaces [15,16,17], and the effect of immobilizing alendronate on the titanium surface to stimulate new bone formation and improve bone-implant integration after implantation has been widely reported [17,18,19,20]. On the titanium surface, alendronate molecules are covalently bound to the TiO_2_ layer via phosphonate groups (-PO_3_H) [15]. At the same time, those unbound phosphonate groups (-PO_3_H), together with the amine groups (-NH_2_) and hydroxyl groups (-OH) of the alendronate molecules, remain free and can participate in the chemical reactions during new bone growth. These reactions include the spontaneous precipitation of different calcium phosphate phases (CaP), including hydroxyapatite (HAp), a naturally occurring form of calcium phosphate that is an essential component for the formation of new bone matrix. The CaP deposit has an important role in initial cell attachment since it can adsorb proteins from the surrounding tissue, promoting cell adhesion and the recruitment of osteogenic cells to the implant surface and facilitating the new bone mineralization process [21,22,23,24]. As known from the literature, the chemical precipitation and nucleation of calcium phosphate (CaP) are strongly influenced by self-assembled monolayers carrying different functional groups [25,26,27,28]. Specifically, different monolayers with phosphonate (-PO_3_H), amino (-NH_2_) or hydroxyl (-OH) groups were investigated [29,30,31,32,33,34,35], and they were found to promote the mineralization process and affect the texture of the CaP deposit. These results sparked our interest in investigating the potential of alendronate coating as a promoter of CaP formation in the contents of the enhanced bioactivity of the underlying titanium.

In our previous publications, the mechanism of the formation of the alendronate coating on the titanium surface and its chemical stability when exposed to artificial saliva was reported in detail [15,16]. Since the alendronate-modified titanium surface has been shown to exhibit good chemical stability, which is the basic prerequisite for its biocompatibility, it was of great importance to investigate bioactivity as an essential property for successful osseointegration. In this study, the bioactivity potential of the alendronate coating was monitored via the spontaneous formation of CaP deposits on the alendronate-coated titanium surface. To the best of our knowledge, a combined experimental and theoretical approach was used for the first time to fully elucidate the formation of CaP. Quantum chemical DFT calculations provided insights into the interactions of the calcium and phosphate ions with alendronate molecules on the titanium surface in the early phase of the CaP nucleation process. The results indicated spontaneous CaP formation, which was confirmed experimentally using scanning electron microscopy (SEM), energy dispersive X-ray spectroscopy (EDS), X-ray diffraction (XRD) and attenuated total reflection Fourier transform infrared spectroscopy (ATR-FTIR). The phase analysis of the CaP deposit confirmed the mixture of the phases, beta-tricalcium phosphate (β-TCP) and calcium-deficient HAp, having beneficial properties from an application point of view.

## 2. Materials and Methods

### 2.1. Quantum Chemical Calculations

Density functional theory (DFT) quantum chemical calculations have been carried out, employing the M06 functional developed by Truhlar’s group [36,37,38]. The 6-31+G(d,p) + LANL2DZ basis set was utilized for geometry optimization. Pople’s 6-31+G(d,p) double-ξ basis set was chosen for the H, C, O, N, Ca, and P atoms, and the LANL2DZ basis set was chosen for the transition metal (Ti) atoms [39]. The vibrational frequency analysis under the harmonic oscillator approximation was used to verify all calculated structures to be true minima on the potential energy surface. In addition, the thermal correction to the Gibbs free energy was derived from the same vibrational analysis. To refine the energy, a highly flexible 6-311++G(2df,2pd) basis set was utilized for H, C, O, N, Ca and P atoms, while the same LANL2DZ ECP-type basis set was used for the titanium atoms. The polarizable continuum solvation model SMD (solvation model based on density) [40] was used to model the solvation effects. The solvent water is represented by a dielectric constant of ε = 78.3553. To account for the specific interaction of the metal cation with the water solvent, the first solvation shell [41], represented by a specific number of water molecules coordinating the calcium ion, is explicitly included in the quantum chemical region, and the remaining bulk solvent is approximated by a polarizable continuum, leading to the cluster continuum method [42]. All calculations were performed with the program package Gaussian 09 (revision D.01) [43].

The topological analysis of the charge density distribution was performed through means of Bader’s quantum theory of atoms in molecules (QTAIM) [44] using the AIMALL [45] program package (version 17.01.25) and utilizing the SMD/M06/6-31+G(d,p) + LANL2DZ wave function obtained from the optimization.

The Gibbs free energy of the interactions, ∆G*_INT_, was calculated according to the formula ∆G*_INT_ = G*_AB_ − G*_A_ − G*_B_, where G*_AB_ is the total free energy of the resulting AB structure and G*_A_ and G*_B_ are the total free energies of the associating units A and B, respectively. A detailed description of the computational modeling can be found in the Supplementary Materials.

### 2.2. Materials, Chemicals and Solutions

The substrates whose surfaces were functionalized were the titanium discs (Ti, 99.9%, *ϕ* = 12 mm, thickness 1.5 mm, Alfa Aesar^®^, Karlsruhe, Germany).

The following chemicals were used as received: sodium alendronate trihydrate (≥97%, Merck Sharp & Dohme, Rahway, NJ, USA), acetone (p.a., Gram-Mol, Zagreb, Croatia) and absolute ethanol (p.a., Gram-Mol, Zagreb, Croatia).

The powder of sodium alendronate trihydrate was dissolved in Milli-Q^®^ water (Millipore, Merck, Darmstadt, Germany) via ultrasonic stirring for 10 min to obtain a 10 mmol dm^−3^ solution. The phosphate-buffered solution (10.0530 g Na_2_HPO_4_·7H_2_O + 1.9501 g NaH_2_PO_4_·2H_2_O were dissolved in 0.5 dm^3^ water; pH = 7.4) was used to prepare an oxide layer on the Ti substrate. The Fusayama artificial saliva, prepared via dissolving 0.4 g NaCl, 0.4 g KCl, 0.6 g CaCl_2_·2H_2_O, 0.58 g Na_2_HPO_4_·2H_2_O and 1 g urea in 1 dm^3^ water [46], was used as a model solution to monitor spontaneous CaP formation. All solutions were prepared with Milli-Q^®^ water.

### 2.3. Functionalisation of the Titanium Samples

The titanium sample covered using electrochemically formed oxide film coated with alendronate (Ti/oxide/alendronate) was prepared.

Prior to modification, the surface of Ti samples was abraded with 240, 500 and 600 grit SiC emery paper, ultrasonically cleaned with absolute ethanol, acetone and Mili-Q^®^ water and dried with nitrogen (99.999%, Messer^®^, Bad Soden, Germany). To create the oxide layer on the Ti surface (Ti/oxide), a potential of 2.5 V vs. Ag|AgCl, 3.0 mol dm^−3^ KCl was applied to the Ti in a phosphate-buffered solution for 5 h. The oxide layer formation was performed on the Ti disc, which was placed in a Teflon holder so that an area of 1 cm^2^ was exposed to the electrolyte. The measurement was performed in a three-electrode cell with the Ti as the working electrode, the Ag|AgCl, 3.0 mol dm^−3^ KCl (*E* = 0.210 V vs. standard hydrogen electrode, SHE) as the reference and the graphite rod as the counter electrode using the Autolab 128N potentiostat/galvanostat (Metrohm Autolab BV, Utrecht, The Netherlands) controlled using Nova 2.1.6^®^ software (Metrohm Autolab BV, Utrecht, The Netherlands). The Ti/oxide samples were then rinsed and dried with nitrogen. Functionalization with an alendronate layer was performed on the Ti sample with oxide layer (Ti/oxide), according to the following procedure. The Ti/oxide samples were immersed in the prepared alendronate solution at 22 ± 1 °C for 24 h. The functionalized samples were then dried at 70 °C for 7 h to ensure the chemical stability of the coating [47]. Samples were then rinsed with Milli-Q^®^ water and absolute ethanol and dried in a nitrogen stream. 

### 2.4. Spontaneous Formation of Calcium Phosphates (CaP) on the Titanium Samples

To investigate potential bioactivity of the Ti/oxide/alendronate sample, samples were immersed in the Fusayama artificial saliva (pH 6.8) at 22 ± 2 °C for a period of 100 days. After this period, white deposits were visible on the surface of the sample, which was characterized in more detail.

### 2.5. Characterisation of the Titanium Samples

The phase composition of the samples was recorded in the 2*θ* range between 10° and 90°, and 45 kV and 40 mA on the Empyrean powder X-ray diffractometer (PXRD) (Malvern Pananalytical B.V., Almelo, The Netherlands) with CuK*α* radiation (0.1542 mm).

Morphology and elemental analysis was performed using the field emission scanning electron microscope (FE-SEM, model JSM-7000F, Jeol Ltd., Tokyo, Japan) in conjunction with the energy dispersive X-ray analyzer EDS/INCA 350, (Oxfore Instruments, High Wycombe, UK). Micrographs were recorded at 5 kV accelerating voltage and 10 mm working distance. The EDS analysis was performed with an accelerating voltage of 10 kV, a working distance of 10 mm, acquisition time of 180 s and a dead time of 16%.

The attenuated total reflectance Fourier transform infrared (ATR-FTIR) spectra were measured with the IRTracer-100 spectrometer (Shimadzu, Kyoto, Japan) during 45 cycles in the range of 4000 to 450 cm^−1^ with a scan resolution of 4 cm^−1^. 

The results shown in this study are the average of three measurements.

## 3. Results and Discussion

### 3.1. The Mechanism of Interaction of Calcium and Phosphate Ions with Ti/Oxide/Alendronate 

To gain insight into the possible interactions between calcium and phosphate ions present in body fluids with Ti/oxide/alendronate at the molecular level, a detailed theoretical study using quantum chemical DFT calculations was performed. The interaction of calcium and phosphate ions with the alendronate-coated surface was analyzed in terms of the interaction pattern and interaction energy. Herein, the aim of the theoretical simulations was not to investigate the entire nucleation route for calcium phosphate, but rather the initial stage of the aggregation of calcium and phosphate ions at the alendronate coating. The hydrogen phosphate anion HPO_4_^2−^ was considered for the simulation as it is the main phosphate species present in the solution at pH = 6.5. Also, it is important to point out that the ion association process between Ca^2+^ and HPO_4_^2−^ occurs immediately at the beginning of the deposition process, forming a CaHPO_4_ ion pair which interacts with alendronate coating. 

Since the TiO_2_ layer is present on the titanium surface, the TiO_2_ nanocluster was selected for the simulation of the metal oxide layer. For the sake of computational efficiency, the (TiO_2_)_10_ nanocluster used by Qu and Kroes [48] was selected to serve for cluster modeling.

Previously reported results for the alendronate–coated titanium surface [15] showed that it is most likely the result of two energetically competing structures, one in which the alendronate molecule is bound to the TiO_2_ surface via both the amine and phosphonate groups, while in the other the alendronate molecule is bound only via the phosphonate group, with the former being slightly more favorable by 3.48 kcal mol^−1^. Therefore, to model the (TiO_2_)_10_-alendronate-CaP structure, both (TiO_2_)_10_–alendronate structures are taken into account.

**Figure 1:**
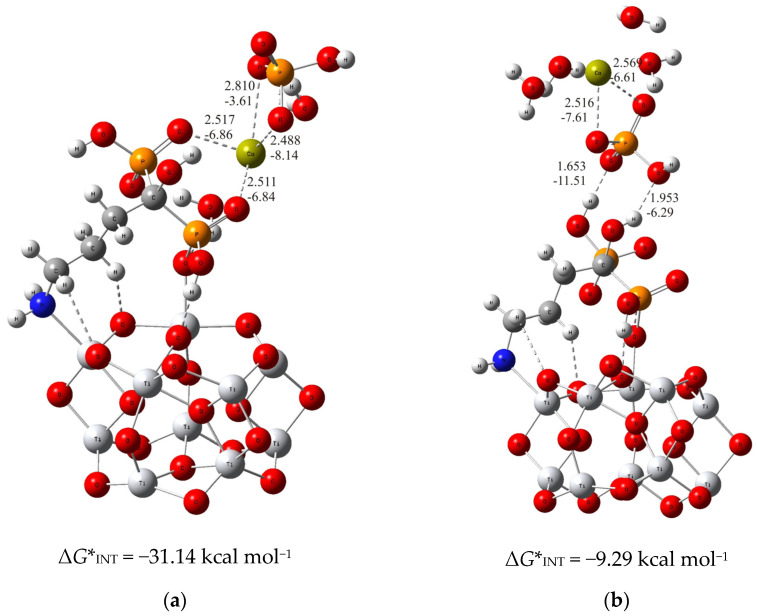
The most stable structures predicted using DFT calculations: (**a**) (TiO_2_)_10_–alendronate–CaP–I and (**b**) (TiO_2_)_10_–alendronate–CaP–II. The bond lengths are given in Å, the bond energies are given in kcal mol^−1^. Ti—light gray, O—red, C—gray, N—blue, P—orange, H—white, Ca—yellow-green.

In the case of the more stable (TiO_2_)_10_–alendronate structure, the formation of the CaP deposit can be initiated by the following two structures. In one, (TiO_2_)_10_–alendronate–CaP–I (Figure 1a), which is more favorable, the calcium ion binds to the two phosphonate groups of the alendronate molecule, releasing the Gibbs free energy of ΔG*_INT_ = −31.14 kcal mol^−1^. 

The interaction between the phosphonate groups and the calcium ion is achieved by two strong coordinate P–O–Ca bonds (*d*_Ca···O_ = 2.517 Å and 2.511 Å; *E*_Ca···O_ = −6.84 kcal mol^−1^ and −6.86 kcal mol^−1^; Figure 1a), where a free electron pair from the oxygen atom of the phosphonate group of the alendronate molecule is involved in the binding with the calcium ion. In addition, the HPO_4_^2−^ ion binds to the calcium ion via Ca–O bonds (*d*_Ca···O_ = 2.810 Å and 2.488 Å; *E*_Ca···O_ = −3.61 kcal mol^−1^ and −8.14 kcal mol^−1^; Figure 1a) in bidentate fashion, and is aligned in the upper part of the (TiO_2_)_10_–alendronate–CaP–I structure. The formed Ca–O bonds are attributed to an ionic interaction type according to ∇^2^*ρ(rc)* > 0 and *H(rc)* > 0 from the topological analysis of the electronic density distribution. In addition, two water molecules are found in the first coordination shell of the Ca^2+^ ion.

When the formation of CaP is accomplished via the phosphate group of the CaHPO_4_ ion pair, the considerably less stable structure ((TiO_2_)_10_–alendronate–CaP–II) with ΔG*_INT_ = −9.29 kcal mol^−1^ is obtained (Figure 1b). In this structure, the phosphate group binds to the alendronate molecule at the phosphonate site via two strong hydrogen O···H–O bonds (*d*_O···H_ = 1.653 Å and 1.953 Å; *E*_O···H_ = −11.51 kcal mol^−1^ and −6.29 kcal mol^−1^; Figure 1b). The remaining two oxygen atoms of the phosphate group coordinate the calcium in a bidentate manner (*d*_Ca···O_ = 2.516 Å and 2.569 Å; *E*_Ca···O_ = −7.61 kcal mol^−1^ and −6.61 kcal mol^−1^; Figure 1b). The critical point of the formed Ca–O bonds was characterized by positive values of the electron density Laplacian, ∇^2^*ρ(rc)* > 0, and by a positive value of the electron energy density, *H(rc)* > 0, which assigns the Ca–O bonds to an ionic interaction type. 

The calculated Gibbs free energies of the interactions for the (TiO_2_)_10_–alendronate–CaP structures selected above point to the spontaneous formation (ΔG*_INT_ < 0) for both structures. However, the binding is found to be much more exergonic in the case of (TiO_2_)_10_–alendronate–CaP–I (Figure 1a), leading to the conclusion that CaP deposition on the alendronate coatings occurs mainly through the calcium (CaHPO_4_)–phosphonate (alendronate) interactions.

**Figure 2:**
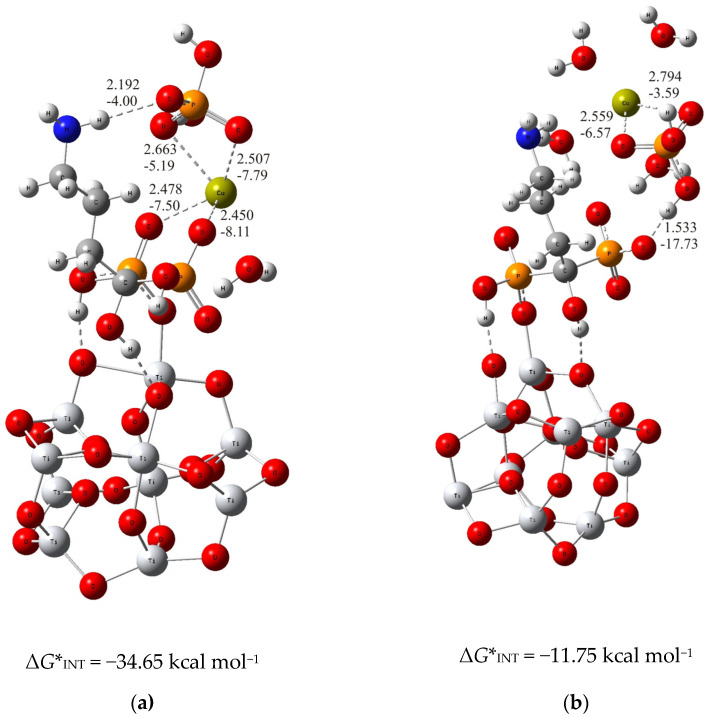
The most stable structures predicted using DFT calculations: (**a**) (TiO_2_)_10_–alendronate–CaP–III and (**b**) (TiO_2_)_10_–alendronate–CaP–IV. The bond lengths are given in Å, the bond energies are given in kcal mol^−1^. Ti—light gray, O—red, C—gray, N—blue, P—orange, H—white, Ca—yellow-green.

For the thermodynamically less stable but highly competitive (TiO_2_)_10_–alendronate structure, two modes of binding of calcium and phosphate ions to alendronate molecule are also obtained (Figure 2). In the one, thermodynamically more stable structure, the calcium and phosphate ions interact with the alendronate molecule via the calcium ion, which binds to two oxygen atoms of the phosphonate group of the alendronate molecule ((TiO_2_)_10_–alendronate–CaP–III, *d*_Ca···O_ = 2.478 Å and 2.450 Å; *E*_Ca···O_ = −7.50 kcal mol^−1^ and −8.11 kcal mol^−1^; Figure 2a). The phosphate ion binds to the calcium ion via two oxygen atoms with d_Ca···O_ = 2.507 Å and 2.663 Å and *E*_Ca···O_ = −7.79 kcal mol^−1^ and −5.19 kcal mol^−1^, respectively (Figure 2a). The additional interaction of the phosphate ion with the amino group is established through the strong O···H–N hydrogen bond (d_O···H_ = 2.192 Å; *E*_O···H_ = −4.00 kcal mol^−1^; Figure 2a). It can be considered, according to the QTAIM analysis, as a partially ionic and partially covalent interaction in accordance with the positive value of Laplacian, ∇^2^*ρ(rc)* > 0, and the negative value of the electron energy density, *H(rc)* < 0, at the bond critical point. The Gibbs free energy of interaction for the structure under consideration is calculated as ΔG*_INT_ = −34.65 kcal mol^−1^. 

In the alternative structure, (TiO_2_)_10_–alendronate–CaP–IV (Figure 2b), the binding of the CaP ion pair via the phosphate group, leads to a less stable structure with a Gibbs free interaction energy of −11.75 kcal mol^−1^. In this structure, the phosphate ion of the CaHPO_4_ ion pair interacts with the phosphonate group of the alendronate molecule, resulting in an anchoring hydrogen O···H–O bond with a corresponding bond length of 1.533 Å and an energy of *E*_O···H_ = −17.73 kcal mol^−1^. The phosphate group coordinates the calcium ion through two bonds with a corresponding bond length of *d*_Ca···O_ = 2.559 Å and 2.794 Å and energies of *E*_Ca···O_ = −6.57 kcal mol^−1^ and −3.59 kcal mol^−1^, respectively (Figure 2b). It is worth mentioning that no interaction of the CaP ion pair with the NH_2_ group of alendronate molecule was found.

In summary, the deposition of calcium and phosphate ions on the alendronate modified titanium oxide surface is governed by Ca^2+^–phosphonate interaction. It is supported by the hydrogen bond between the phosphate group of CaP and the amino group of the alendronate molecule, as found in the structure (TiO_2_)_10_–alendronate–CaP–III. Those interactions ensure a thermodynamically strong favorable binding pattern with a released Gibbs free interaction energy of ΔG*_INT_ = −34.65 kcal mol^−1^. 

**Figure 3:**
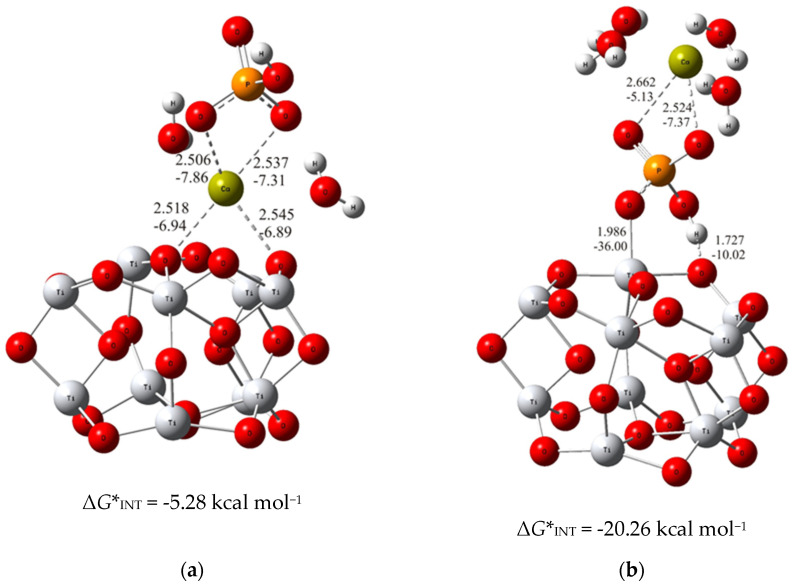
The most stable structures predicted using DFT calculations: (**a**) (TiO_2_)_10_–CaP–I and (**b**) (TiO_2_)_10_–CaP–II. The bond lengths are given in Å, the bond energies are given in kcal mol^−1^. Ti—light gray, O—red, C—gray, P—orange, H—white, Ca—yellow-green.

To put the above results in perspective, the DFT calculations of the interactions of bare TiO_2_ clusters with calcium phosphate ions were carried out. The most stable structure for each of the considered binding modes (analogous to the previous ones with alendronate coating) were determined and demonstrated in Figure 3. 

As can be seen from the DFT results, the binding established through the interaction of the phosphate ion of CaP (ΔG*_INT_ = −20.26 kcal mol^−1^; Figure 3b) with the TiO_2_ surface is more exergonic than the binding achieved through the Ca^2+^ – O_(TiO2)10_ (ΔG*_INT_ = −5.28 kcal mol^−1^; Figure 3a) interactions. However, compared to the most favorable binding of calcium phosphate ions to the alendronate modified titanium oxide surface, it appeared significantly less exergonic (Δ(ΔG*_INT_) = 14.39 kcal mol^−1^). These findings clearly indicate the positive effects of alendronate coating on the formation of CaP which is fully in line with the experimental observations.

### 3.2. Experimental Evidence for the Spontaneous Formation of Calcium Phosphates (CaP) on the Ti/oxide/Alendronate Surface

Since the DFT results clearly showed that the spontaneous formation of CaP on the alendronate-modified Ti/oxide surface is possible, experimental evidence was obtained using SEM, EDS, ATR-FTIR and XRD techniques. The prepared Ti/oxide/alendronate samples were immersed for 100 days in Fusayama artificial saliva solution as simulated body fluid to test alendronate coating’s bioactivity on the basis of monitoring the spontaneous CaP deposit formation.

#### Morphology, Chemical and Phase Analysis of Ti/Oxide/Alendronate Samples after 100 Days Immersion in Artificial Saliva

**Figure 4:**
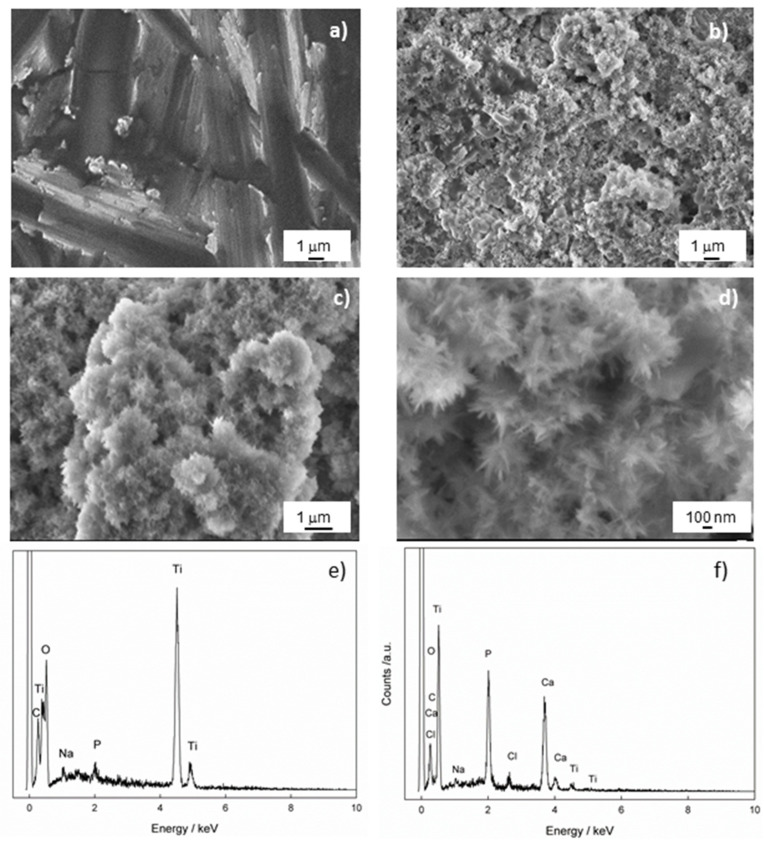
The FE-SEM images of Ti/oxide/alendronate (**a**) before and (**b**–**d**) after immersion in artificial saliva. The images were taken at (**a**,**b**) 5000×, (**c**) 10,000× and (**d**) 33,000× magnification. The chemical composition of the Ti/oxide/alendronate sample (**e**) before and (**f**) after immersion in artificial saliva.

SEM measurements were performed to gain insight into the morphology of the Ti/oxide/alendronate sample before and after immersion in the artificial saliva for 100 days. The results are shown in Figure 4. The inhomogeneous-layered structure is evident for the Ti/oxide/alendronate surface (Figure 4a), as a consequence of the presence of electrochemically prepared oxide film on the Ti surface. It is well-known that the alendronate coating has no significant effect on the morphology due to its low thickness (one monolayer) [47].

The immersion of the sample in the artificial saliva over a period of 100 days changed the morphology of the Ti/oxide/alendronate surface significantly and resulted in the spontaneous formation of agglomerates over almost the entire surface (Figure 4b,c). A deeper look into the spontaneously formed deposit (Figure 4d) revealed a flower agglomerate consisting of nanoneedles. The morphology obtained is one of the possible forms of CaP structures such as nanosheets, microrods, and microplates, which are influenced by the precipitation conditions (temperature, pH, reactants and their concentrations) [49,50]. Nanoneedles are also observed under similar pH conditions [50,51,52]. 

To check the chemical composition of the Ti/oxide/alendronate sample before and after immersion in artificial saliva, the samples were analyzed using EDS. The results are shown in Table 1 and Figure 4e,f, and indicate that the alendronate coating (Na and P were detected) was present over the oxide layer on the titanium before immersion in artificial saliva. As can be seen after 100 days of immersion, the elements Na, Ca, P and Cl were detected on the surface of the sample, whereby the amount of the element P increased significantly and the other elements Ca and Cl were incorporated into the structure of the deposit (Table 1). The Ca/P atomic ratio is 1.51 and may indicate the presence of calcium-deficient hydroxyapatite (CDHAp) [53]. Furthermore, due to the presence of the elements Cl and Na, it can be concluded that the apatite formed is biocompatible, closely resembling the natural bone composition, which could have a positive effect on bone metabolism [54].

It should be emphasized that atmospheric carbon contamination is possible, which could influence the EDS results. The prompt gamma-ray activation analysis (PGAA) can be useful for the accurate determination of elemental composition, as shown by A. Nespoli et al. [55]. 

**Figure 5:**
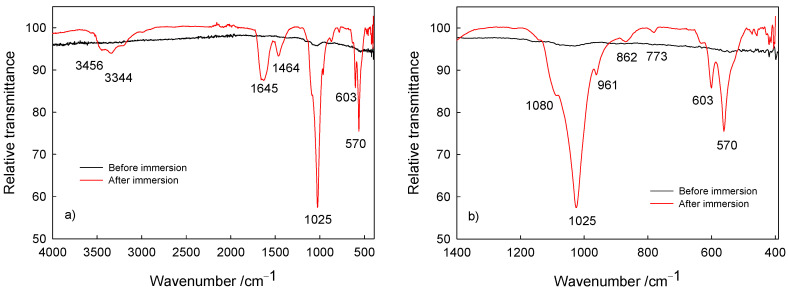
The ATR-FTIR of Ti/oxide/alendronate before and after immersion in artificial saliva: (**a**) wide range spectra and (**b**) P–O bands in the fingerprint region of the spectra.

The surface characterization (composition) of Ti/oxide/alendronate samples before and after immersion was performed using ATR-FTIR (Figure 5). Prior to sample immersion, weak bands around 1000 cm^–1^ are visible characteristics for the P–O and P=O bands present in the alendronate molecule [16,56,57]. On the other hand, after immersion bands were detected in the spectral range from 900 to 1100 cm^–1^, typical for the symmetric and asymmetric P–O stretching modes in the phosphate group [58,59,60]. The noticeable strong bands are indicative of phosphate layer deposits; i.e., dominant ν_3_ PO_4_^3–^ absorption bands in the range 1080–1000 cm^–1^ (P–O asymmetric stretching vibrations), the ν_1_ PO_4_^3–^ band at 961 cm^–1^ (P–O symmetric stretching vibrations) and the bending mode bands (O–P–O vibrations) in range from 560 to 630 cm^–1^ [58,59,60]. The visible band at 1645 cm^–1^ is assigned to the ν_2_ bending mode of adsorbed water associated with the hydroxyapatite phase [58], accompanied with bands at 3456 and 3344 cm^–1^ typical for the hydroxyl stretching present in hydroxyapatite [59]. 

Additionally, the bands at 1464, 862 and 774 cm^–1^ are attributed to the ν_1_ CO_3_^2–^ band (asymmetric stretching mode of carbonate group), the ν_2_ CO_3_^2–^ band (symmetric stretching mode of carbonate group) and the ν_4_ CO_3_^2–^ band (asymmetric stretching mode of carbonate group) and point to the carbonate content present in the calcium phosphate deposit [58,59], which is due to the atmospheric conditions during spontaneous deposition. Carbonate ions replace hydroxyl or phosphate ions in the hydroxyapatite coating, resulting in the formation of calcium-deficient hydroxyapatite with a decreased Ca/P ratio in comparison to the Ca/P ratio of hydroxyapatite [21]. This was the case for the deposit investigated, with the Ca/P ratio of 1.51. Hence, the results obtained are in accordance with the SEM/EDS results discussed previously in this section. The detected carbonate content should not be considered detrimental or unwanted since it allows for a closer resemblance to the natural bone composition [22,61].

The obtained ATR-FTIR spectra confirm the spontaneous formation of a calcium phosphate deposit on the Ti/oxide/alendronate sample during immersion in the artificial saliva solution, corroborating the enhanced bioactivity of the titanium implant material upon alendronate coating formation.

**Figure 6:**
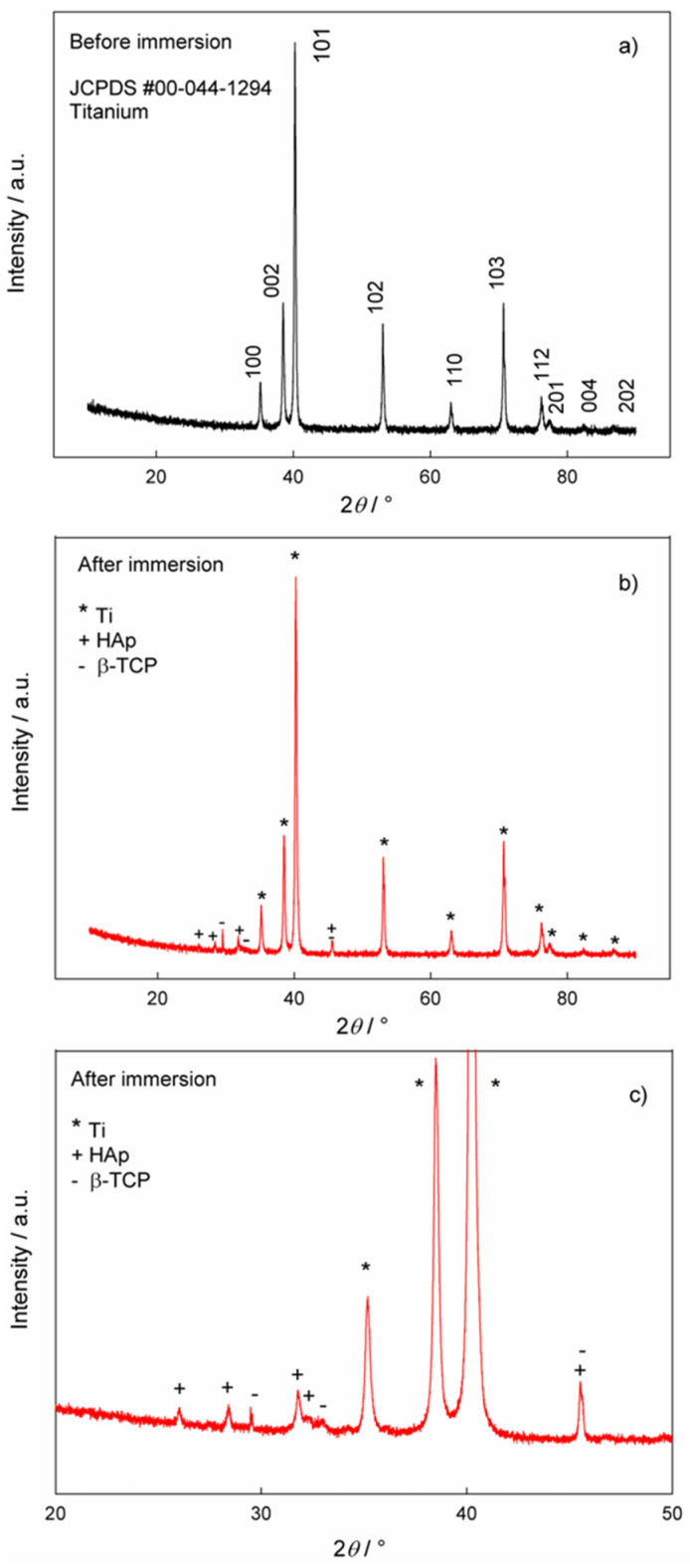
The XRD patterns of the Ti/oxide/alendronate sample (**a**) before and (**b**,**c**) after immersion in artificial saliva. (**c**) Pattern from figure (**b**) in the narrower 2*θ* region, 20–50°.

The phase analysis of the samples before and after immersion in artificial saliva solution was performed using XRD and the results are shown in Figure 6. Figure 6a shows the XRD data for the Ti/oxide/alendronate before immersion in artificial saliva solution. The comparison of the experimental data with the reference card for titanium (JCPDS #00-044-1294) [62] clearly shows the dominance of the titanium substrate as a single phase. Since the electrochemically formed oxide layer is obviously very thin, the oxide phase was not detected in the XRD pattern.

The XRD pattern of the Ti/oxide/alendronate sample after immersion in artificial saliva solution contains characteristic peaks for hydroxyapatite (HAp, JCPDS # 00-009-0432) [53] and beta-tricalcium phosphate (β-TCP, JCPDS # 00-009-0169) [21,63], (Figure 6b,c). The peaks of the titanium substrate are also visible. The results are in accordance with the ATR-FTIR results. The experimental conditions (artificial saliva with a pH of 6.5, room temperature and 100 days immersion) lead to the formation of a mixture of β-TCP and HAp in the form of floral deposits of nanoneedles. The result is consistent with the EDS results, where the Ca/P ratio also indicates the formation of HAp, i.e., calcium-deficient HAp. The formed mixture of HAp and β-TCP can be very useful from an application point of view, as this combination is an important material for tissue engineering and a process of bone formation [64].

In summary, the formation of calcium-deficient hydroxyapatite was spontaneously induced on the titanium covered with electrochemically prepared oxide film and was subsequently modified by the alendronate coating, as was validated using XRD, EDS and ATR-FTIR characterization methods. Spontaneous formation points to the bioactivity of the alendronate coating, resulting in the development of a calcium phosphate phase with beneficial properties. Since, the presence of hydroxyapatite plays a crucial role in various phases of new bone growth, including cell adhesion, the bone formation phase, also serving as a scaffold for new bone deposition, the mineralization phase and bone remodeling, its presence facilitates and is the essential for the successful implant integration [21,22,23,24]. 

## 4. Conclusions

In this study, the spontaneous calcium phosphate deposition onto an alendronate modified TiO_2_ surface was investigated both in silico by means of DFT quantum chemical calculations and in vitro by a simple immersion procedure in artificial saliva. 

The DFT results showed that the molecular interactions between the alendronate–coated Ti/oxide surface and the calcium and phosphate ions were spontaneous according to the calculated negative Gibbs free interaction energy. It has been shown that the deposition of calcium and phosphate ions on the alendronate-modified titanium surface is determined by the interaction of Ca^2+^ with the phosphonate group (-PO_3_H) of the alendronate molecule and is strongly supported by the O···H–N hydrogen bonding between the phosphate (HPO_4_^2−^) and the amino group (-NH_2_) of the alendronate molecule. The formation of the most stable (TiO_2_)_10_–alendronate–CaP structure proves to be a highly exergonic process with a calculated Gibbs free interaction energy of ΔG*_INT_ = −34.65 kcal mol^−1^. 

The results of scanning electron microscopy, energy dispersive X-ray spectroscopy and attenuated total reflectance Fourier transform infrared spectroscopy confirmed spontaneous calcium phosphate deposition on the Ti/oxide/alendronate surface after 100 days of exposure of the sample to artificial saliva. The spontaneously formed deposit is a mixture of two phases, beta-tricalcium phosphate and calcium-deficient hydroxyapatite, according to the X-ray diffraction phase analysis. The presence of trace elements (Na, Cl) and carbonate ions in the hydroxyapatite structure, detected using energy dispersive X-ray spectroscopy and attenuated total reflectance Fourier transform infrared spectroscopy, indicates the biological hydroxyapatite, which is also confirmed by the Ca/P ratio of 1.51 (energy dispersive X-ray spectroscopy).

These results indicate the potential bioactivity of functionalized titanium and provide fundamental information useful for the development of dental implants with improved osseointegrity. For further clinical testing, biological studies in a medium similar to the complex oral environment followed by in vivo studies are required.

## Figures and Tables

**Table 1:** Chemical composition of the Ti/oxide/alendronate sample before and after the immersion in artificial saliva (the average of three measurements).

Element, at. %							
Sample	Ti K	C K	O K	Na K	P K	Ca K	Cl K
Before immersion	40.76	12.18	45.16	0.99	0.91	/	/
After immersion	1.41	16.68	54.22	0.49	10.41	15.74	1.05

## Data Availability

The original contributions presented in the study are included in the article/Supplementary Materials, further inquiries can be directed to the corresponding authors.

## Supplementary Materials

The following supporting information can be downloaded at: https://www.mdpi.com/article/10.3390/ma17112703/s1, Computational modeling, Figure S1: Optimized structures of the selected systems (bond distances in Å, bond energies in kcal mol^−1^), Table S1: Formation of CaP layer. Standard state (1M) free energies of interaction Δr*G**_INT_ computed by using the SMD solvation model at the M06/6-311++G(2df,2pd) + LANL2DZ// M06/6-31+G(d,p) + LANL2DZ level of theory (in kcal mol^−1^), Table S2: Total electronic energy, *E*^Tot^_soln_, obtained at the SMD/M06/6-311++G(2df,2pd) + LANL2DZ//SMD/M06/6-31+G(d,p) + LANL2DZ level of theory, thermal correction to the Gibbs free energy, Δ*G**_VRT,soln_, obtained at the SMD/M06/6-31+G(d,p) + LANL2DZ level of theory, and total free energy, *G**_X_, (*G**_X_ = *E*^Tot^_soln_ + Δ*G**_VRT,soln_) in water media of the investigated species (all energies in hartree), Table S3: Bond lengths (d), energies (E) and QTAIM properties of the selected bonds in the investigated systems, Cartesian coordinates of the calculated systems. Refs. [65,66,67,68,69,70,71,72,73,74,75] are cited in the Supplementary Materials.
