# Supplementary material for: Alendronate as Bioactive Coating on Titanium Surfaces: An Investigation of CaP–Alendronate Interactions
Source: Materials (Basel). 2024 Jun 3;17(11):2703. doi: 10.3390/ma17112703 (PMC11173899; doi:10.3390/ma17112703)
Supplement: Supplementary file 1 [file materials-17-02703-s001.zip › materials-2980838-supplementary.pdf]

Supporting Information

for

Alendronate as bioactive coating on titanium surfaces: An investigation of CaP–alendronate interactions

Ines Despotović [1,*], Željka Petrović [2,*], Jozefina Katić [3] and Dajana Mikić [3]

Address:

[1*] Division of Physical Chemistry, Ruđer Bošković Institute, Bijenička cesta 54, 10002 Zagreb, Croatia; Ines.Despotovic@irb.hr

[2*] Division of Materials Chemistry, Ruđer Bošković Institute, Bijenička cesta 54, 10002 Zagreb, Croatia; Zeljka.Petrovic@irb.hr

[3] Department of Electrochemistry, Faculty of Chemical Engineering and Technology, University of Zagreb, Marulićev trg 19, 10000 Zagreb, Croatia; jkatic@fkit.hr; dmikic@fkit.unizg.hr

Computational modeling

All calculations were performed by means of quantum chemical calculations at the density functional theory (DFT) level using the Gaussian 09 program (revision D.01) [1].

The M06 functional designed by the Truhlar's group, which provides very accurate thermodynamic parameters, being particularly successful in nonbonding interactions treatment, has been selected [2-4]. The 6-31+G(d,p) + LANL2DZ mixed basis set has been utilized. The Pople's 6-31+G(d,p) double-ξ basis set was chosen for O, H, C, N, P, Ca atoms and the LANL2DZ basis (LANL2 pseudopotential for inner electrons and its associated double-ξ basis set (DZ)) was used for the transition-metal (Ti) atoms [5]. This gave rise to the M06/6-31+G(d,p) + LANL2DZ model utilized for geometry optimization which has been frequently used for studies of transition-metal containing systems. The geometric structures of the molecules were optimized by minimizing energies with respect to the geometrical parameters without imposing any molecular symmetry constraints and using a tight convergence condition. The Berny algorithm using redundant internal coordinates was employed. Frequency calculations were made under the harmonic approximation on all the optimized structures at the same level of theory with no scaling in order to confirm that the structures correspond to the true minima, meaning that no imaginary frequencies were present, as well as to extract thermal Gibbs free energy corrections. The final single point energies were obtained using a highly flexible 6-311++G(2df,2pd) basis set for the O, H, C, N, P, Ca atoms, while the same LANL2DZ ECP type basis set for titanium atoms was employed.

The self- consistent field (SCF) calculations were conducted under a tight condition imposing the threshold value of 10^{-8} hartree to total energy difference during the iteration process. The integration grid was set to FineGrid having 75 radial shells and 302 angular points per shell. The 2-electron integral accuracy was set to 10^{-13}. The FoFCou algorithm with NoSymm option was utilized. All the geometry optimizations, frequency calculations and single point energy evaluations were performed by taking solvent effects into account. To evaluate the bulk solvent effects (water, ε = 78.3553 at 25°), the implicit SMD polarizable continuum solvation model [6] has been employed. It represents a very practical approach to simulate the solvation environment and determine the effect of the medium on the structure and stability of solutes in a solution. To account for the specific interaction of metal cation with water solvent, the first solvation shell [7], represented by a specific number of water molecules coordinating calcium ion was explicitly included in the quantum chemical region.

The conformational space was manually sampled for the $(TiO_2)_{10}$—alendronate—CaP and $(TiO_2)_{10}$—CaP taking into account various donor and acceptor sites of the $(TiO_2)_{10}$—alendronate or $(TiO_2)_{10}$ and CaP species which was modelled by $CaHPO_4$ ion pair. The $(TiO_2)_{10}$ clusters were employed as proposed by Allard et al. [9] and Qu and Kroes [10]. The alendronate molecule was taken in the dianionic form. The $CaHPO_4$ ion pair was formed from the hydrated Ca^{2+} ion (six water molecules have been included into the first solvation shell) and HPO_4^{2-} phosphate ion. During the optimization process the geometry of the $(TiO_2)_{10}$ cluster within the investigated systems was kept fixed.

The interaction Gibbs free energies, ΔG^*_{INT} were computed as the difference between the total free energy (G^*_{ABC}) of the $(TiO_2)_{10}$—alendronate—CaP ($(TiO_2)_{10}$—CaP) structure and the sum of the total free energies ($G^*_A + G^*_B + G^*_C$) of the associating units A, B and C ($(TiO_2)_{10}$—alendronate ($(TiO_2)_{10}$), hydrated Ca^{2+} ion and HPO_4^{2-} phosphate ion) using the supramolecular approach:

$$\Delta G^*_{INT,ABC} = G^*_{AB} - G^*_A - G^*_B - G^*_C \quad (1)$$

The species total free energy in the liquid was calculated using the expression:

$$G^*_X = E^{Tot}_{soln} + \Delta G^*_{VRT,soln} \quad (2)$$

where E^{Tot}_{soln} corresponds to the basic energy of a density functional theory calculation using the SMD model, while $\Delta G^*_{VRT,soln}$ encompasses vibrational, rotational and translational contribution to the solution

free energy, being computed by applying the ideal gas partition functions to the frequencies calculated in the dielectric medium and the 1M standard state. A more negative value of the binding energy implied the more stable formed species. No BSSE correction of binding energies has been applied.

The topological analysis of the charge density distribution using the Bader's quantum theory of atoms in molecules (QTAIM) [11~~10~~] was performed by employing AIMALL software package [12~~11~~] using the SMD/M06/6-31+G(d,p) + LANL2DZ wave function obtained from optimization. Within the QTAIM analysis the electron density was analysed for two major characteristics: (a) the existence of critical points (CPs), where electron density exhibits maximum, minimum, or a saddle point in space, and (b) for the bond paths [13~~12~~], the maximum electron density line connecting two interacting atoms in the energetic minimum structure (the two atoms are bonded). The point of the electron density minimum value along that line called the bond critical point (BCP) and values of topological parameters, like electron density $\rho(r_c)$, Laplacian $\nabla^2\rho(r_c)$, electronic kinetic energy $G(r_c)$, electronic potential energy density $V(r_c)$, total energy density $H(r_c)$ at that point explain interatomic interaction features. $\nabla^2\rho(r_c) < 0$ indicates locally concentrated charge density, while locally depleted charge density is indicated by $\nabla^2\rho(r_c) > 0$. The chemical bond nature can be described qualitatively concerning signs and values of the electron density Laplacian $\nabla^2\rho(r_c)$ and the electron energy density $H(r_c)$ at the bond critical point according to following criteria. The interactions characterized by $\nabla^2\rho(r_c) < 0$ and $H(r_c) < 0$ (shared interaction) are characteristic for weakly polar and nonpolar covalent bonds. On the other hand, $\nabla^2\rho(r_c) > 0$ and $H(r_c) > 0$ (closed shell interactions) point to ionic bonds, weak hydrogen bonds, and van der Waals interactions. The intermediate interactions which include strong hydrogen bonds and most of the coordinate bonds are characterized by $\nabla^2\rho(r_c) > 0$ and $H(r_c) < 0$ [14,15]. A very high negative value of the $\nabla^2\rho(r_c)$ is an indication of a strong covalent bond, while a high positive value corresponds to a strong noncovalent bond. The energies of the coordinate bonds and of other intermolecular hydrogen bonds have been calculated by the Espinosa's equation [16]:

$$E = 0.5\ V(r_c) \tag{3}$$

where E is the bond energy (a.u.), and $V(r_c)$ is potential energy density (a.u.) at the corresponding critical point. The Espinosa's relationship is widely used for the energy estimation of different types of hydrogen [17,18], van der Waals [19], coordinate [20,21], and homopolar bonds [22].

References

1. Gaussian 09, Revision D.01, Frisch, M.J.; Trucks, G.W.; Schlegel, H.B.; Scuseria, G.E.; Robb, M.A.; Cheeseman, J.R.; Scalmani, G.; Barone, V.; Mennucci, B.; Petersson, G.A.; Nakatsuji, H.; Caricato, M.; Li, X.; Hratchian, H.P.; Izmaylov, A.F.; Bloino, J.; Zheng, G.; Sonnenberg, J.L.; Hada, M.; Ehara, M.; Toyota, K.; Fukuda, R.; Hasegawa, J.; Ishida, M.; Nakajima, T.; Honda, Y.; Kitao, O.; Nakai, H.; Vreven, T.; Montgomery, Jr., J.A.; Peralta, J. E.; Ogliaro, F.; Bearpark, M.; Heyd, J.J.; Brothers, E.;. Kudin, K.N.; Staroverov, V.N.; Kobayashi, R.; Normand, J.; Raghavachari, K.; Rendell, A.; Burant, J.C.; Iyengar, S.S.; Tomasi, J.; Cossi, M.; Rega, N.; Millam, J.M.; Klene, M.; Knox, J.E.; Cross, J.B.; Bakken, V.; Adamo, C.; Jaramillo, J.; Gomperts, R.; Stratmann, R.E.; Yazyev, O.; Austin, A.J.; Cammi, R.; Pomelli, C.; Ochterski, J.W.; Martin, R.L.; Morokuma, K.; Zakrzewski, V.G.; Voth, G.A.; Salvador, P.; Dannenberg, J.J.; Dapprich, S.; Daniels, A.D.; Farkas, Ö.; Foresman, J.B.; Ortiz, J.V.; Cioslowski, J.; Fox, D.J. Gaussian, Inc., Wallingford CT, 2013.
2. Zhao, Y.; Thrular, D.G. The M06 suite of density functionals for main group thermochemistry, thermochemical kinetics, noncovalent interactions, excited states, and transition elements: two new functionals and systematic testing of four M06-class functionals and 12 other functionals. *Theor. Chem. Acc.* **2008**, *120*, 215-241., doi: 10.1007/s00214-007-0310-x.
3. Zhao, Y.; Truhlar, D.G. Density Functionals with Broad Applicability in Chemistry. *Acc. Chem. Res.* **2008**, *41*, 157–167., doi:10.1021/ar700111a.

4. Zhao, Y.; Truhlar, D.G. Density Functional Theory for Reaction Energies: Test of Meta and Hybrid Meta Functionals, Range-Separated Functionals, and Other High-Performance Functionals. *J. Chem. Theory Comput.* **2011**, *7*, 669–676., doi:10.1021/ct1006604.
5. Wadt, W.R.; Hay, P.J. Ab initio effective core potentials for molecular calculations. Potentials for main group elements Na to Bi. *J. Chem. Phys.* **1985**, *82*, 284–298., doi:10.1063/1.448800.
6. Marenich, A.V.; Cramer, C.J.; Truhlar, D.G. Universal Solvation Model Based on Solute Electron Density and on a Continuum Model of the Solvent Defined by the Bulk Dielectric Constant and Atomic Surface Tensions. *J. Phys. Chem. B* **2009**, *113*, 6378–6396., doi.org/10.1021/jp810292n.
7. Srinivasa Rao, J.; Dinadayalane, T. C.; Leszczynski, J.; Sastry, N. Comprehensive Study on the Solvation of Mono- and Divalent Metal Cations: Li^+, Na^+, K^+, Be^{2+}, Mg^{2+} and Ca^{2+}. *J. Phys. Chem. A* **2008**, *112*, 50, 12944–12953., doi.org/10.1021/jp8032325
8. Petrović, Ž.; Šarić, A.; Despotović, I.; Katić, J.; Peter, R.; Petravić, M.; Petković, M. A New Insight into Coating's Mechanism Between TiO_2 and Alendronate on Titanium Dental Implant. *Materials* **2020**, *13*, 3220-3235., doi.org/10.3390/ma13143220
9. Allard, M.M.; Merlos, S.N.; Springer, B.N.; Cooper, J.; Zhang, G.; Boskovic, D.S.; Kwon, S.R.; Nick, K.E.; Perry, C.C. Role of TiO_2 Anatase Surface Morphology on Organophosphorus Interfacial Chemistry. *J. Phys. Chem. C* **2018**, *122*, 29237–29248., doi: 10.1021/acs.jpcc.8b08641.
10. Qu, Z.; Kroes, G.-J. Theoretical Study of Stable, Defect-Free $(TiO_2)n$ Nanoparticles with n = 10−16. *J. Phys. Chem. C* **2007**, *111*, 16808–16817., doi: 10.1021/jp073988t.
11. Bader, R.F.W. *Atoms in Molecules: A Quantum Theory*, Oxford University Press, Oxford, New York, USA, 1994.
12. Keith, T.A. *AIMAll (Version 17.01.25)*, TK Gristmill Software, Overland Park KS, USA, 2017 (aim.tkgristmill.com)
13. Bader, R.F.W. A Bond Path: A Universal Indicator of Bonded Interactions. *J. Phys. Chem. A.* **1998**, *102*, 7314–7323., doi:10.1021/jp981794v.
14. Bader, R.F.W.; Essén, H. The characterization of atomic interactions. *J. Chem. Phys.* **1984**, *80*, 1943–1960., doi:10.1063/1.446956.
15. Cremer, D.; Kraka, E. A Description of the Chemical Bond in Terms of Local Properties of Electron Density and Energy. *Croat. Chem. Acta* **1984**, *57*, 1259–1281.
16. Espinosa, E.; Molins, E.; Lecomte, C. Hydrogen bond strengths revealed by topological analyses of experimentally observed electron densities. Chem. Phys. Lett. 1998, 285, 170–173., doi:10.1016/S0009-2614(98)00036-0.
17. Espinosa, E.; Alkorta, I.; Rozas, I.; Elguero, J.; Molins, E. About the evaluation of the local kinetic, potential and total energy densities in closed-shell interactions. *Chem. Phys. Lett.* **2001**, *336*, 457–461., doi:10.1016/S0009-2614(01)00178-6.
18. Borissova, A.O.; Antipin, M.Y.; Karapetyan, H.A.; Petrosyan, A.M.; Lyssenko, K.A. Cooperativity effects of H-bonding and charge transfer in an L-nitroarginine crystal with Z′ > 1. *Mendeleev Commun.* **2010**, *20*, 260–262., doi:10.1016/j.mencom.2010.09.006.
19. Baryshnikov, G.V.; Minaev, B.F.; Minaeva, V.A.; Nenajdenko, V.G. Single crystal architecture and absorption spectra of octathio[8]circulene and *sym*-tetraselenatetrathio[8]circulene: QTAIM and TD-DFT approach. *J. Mol. Model.* **2013**, *19*, 4511–4519. , doi:10.1007/s00894-013-1962-1.
20. Baryshnikov, G.V.; Minaev, B.F.; Korop, A.A.; Minaeva, V.A.; Gusev, A.N. Structure of zinc complexes with 3-(pyridin-2-yl)-5-(arylideneiminophenyl)-1HH-1,2,4-triazoles in different tautomeric forms: DFT and QTAIM study. *Russ. J. Inorg. Chem.* **2013**, *58*, 928–934., doi:10.1134/S0036023613080032.
21. Shahangi, F.; Chermahini, A.N.; Farrokhpour, H.; Teimouri, A. Selective complexation of alkaline earth metal ions with nanotubular cyclopeptides: DFT theoretical study. *RSC Adv.* **2014**, *5*, 2305–2317., doi:10.1039/C4RA08302D.
22. Puntus, L.N.; Lyssenko, K.A.; Antipin, M.Y.; Bünzli, J.C.G. Role of Inner- and Outer-Sphere Bonding in the Sensitization of EuIII-Luminescence Deciphered by Combined Analysis of Experimental Electron Density Distribution Function and Photophysical Data. *Inorg. Chem.* **2008**, *47*, 11095–11107., doi:10.1021/ic801402u.

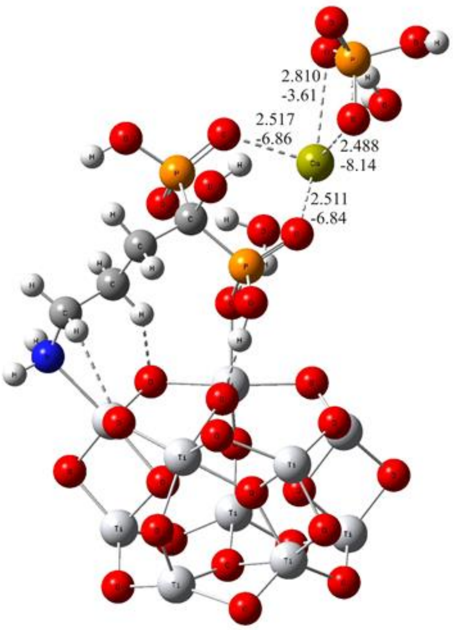

(TiO$_2$)$_{10}$−alendronate−CaP−I

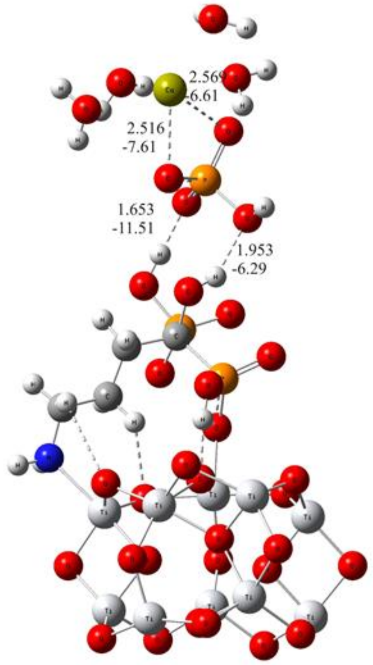

(TiO$_2$)$_{10}$−alendronate−CaP−II

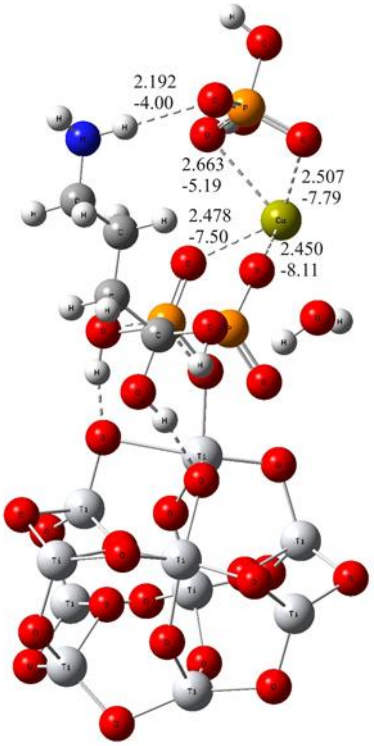

(TiO$_2$)$_{10}$−alendronate−CaP−III

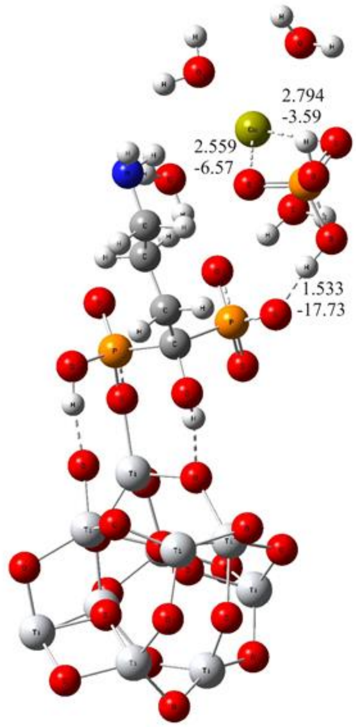

(TiO$_2$)$_{10}$−alendronate−CaP−IV

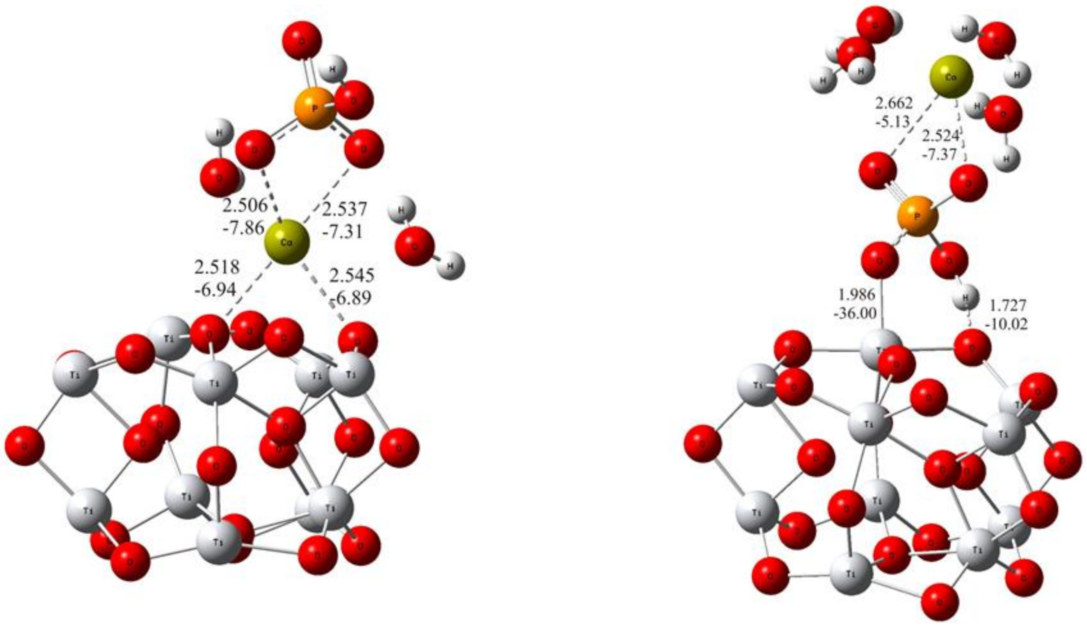

$(TiO_2)_{10}$−CaP−I $(TiO_2)_{10}$−CaP−II

Figure S1: Optimized structures of the selected systems (bond distances in Å, bond energies in kcal mol^{-1})

Table S1. Formation of CaP layer. Standard state (1M) free energies of interaction $\Delta_r G^*_{INT}$ computed by using the SMD solvation model at the M06/6-311++G(2df,2pd) + LANL2DZ// M06/6-31+G(d,p) + LANL2DZ level of theory (in kcal mol^{-1}).

**Interaction**	$\Delta_r G^*_{INT}$
$(TiO_2)_{10}$–alendronate–I[a] + $[Ca(H_2O)_6]^{2+}$ + HPO_4^{2-} → $(TiO_2)_{10}$–alendronate–CaP–I + $4H_20$	-31.14
$(TiO_2)_{10}$–alendronate–I + $[Ca(H_2O)_6]^{2+}$ + HPO_4^{2-} → $(TiO_2)_{10}$–alendronate–CaP–II + $2H_20$	-9.29
$(TiO_2)_{10}$–alendronate–II[b] + $[Ca(H_2O)_6]^{2+}$ + HPO_4^{2-} → $(TiO_2)_{10}$–alendronate–CaP–III + $4H_20$	-34.65
$(TiO_2)_{10}$–alendronate–II + $[Ca(H_2O)_6]^{2+}$ + HPO_4^{2-} → $(TiO_2)_{10}$–alendronate–CaP–IV + $2H_20$	-11.75
$(TiO_2)_{10}$ + $[Ca(H_2O)_6]^{2+}$ + HPO_4^{2-} → $(TiO_2)_{10}$–CaP–I + $4H_20$	-5.28
$(TiO_2)_{10}$ + $[Ca(H_2O)_6]^{2+}$ + HPO_4^{2-} → $(TiO_2)_{10}$–CaP–II + $2H_20$	-20.26

[a]The more stable structure of the $(TiO_2)_{10}$–alendronate where the binding of the alendronate molecule to the $(TiO_2)_{10}$ cluster is accomplished through the phosphonate and amino group [b]The thermodynamically competitive structure of the $(TiO_2)_{10}$–alendronate where the binding of the alendronate molecule to the $(TiO_2)_{10}$ cluster is accomplished only through the phosphonate group.

Table S2. Total electronic energy, E^{Tot}_{soln}, obtained at the SMD/M06/6-311++G(2df,2pd) + LANL2DZ//SMD/M06/6-31+G(d,p) + LANL2DZ level of theory, thermal correction to the Gibbs free energy, $\Delta G^{*}_{VRT,soln}$, obtained at the SMD/M06/6-31+G(d,p) + LANL2DZ level of theory, and total free energy, G^{*}_{X}, ($G^{*}_{X} = E^{Tot}_{soln} + \Delta G^{*}_{VRT,soln}$) in water media of the investigated species (all energies in hartree).

Species	E^{Tot}_{soln}	$\Delta G^{*}_{VRT,soln}$	G^{*}_{X}
$(TiO_2)_{10}$	-2087.42891	-0.03560	-2087.46451
$(TiO_2)_{10}$−alendronate−I	-3511.01334	0.13054	-3510.88280
$(TiO_2)_{10}$−alendronate−II	-3511.00534	0.12685	-3510.87849
$[Ca(H_2O)_6]^{2+}$	-1136.14463	0.09915	-1136.04548
HPO_4^{2-}	-643.28858	-0.00311	-643.29169
H_2O	-76.43583	0.00298	-76.43285
$(TiO_2)_{10}$−alendronate−CaP−I	-4984.73125	0.19306	-4984.53819
$(TiO_2)_{10}$−alendronate−CaP−II	-5137.60000	0.23091	-5137.36909
$(TiO_2)_{10}$−alendronate−CaP−III	-4984.72611	0.18663	-4984.53948
$(TiO_2)_{10}$−alendronate−CaP−IV	-5137.60604	0.23734	-5137.36870
$(TiO_2)_{10}$−CaP−I	-3561.10132	0.02261	-3561.07871
$(TiO_2)_{10}$−CaP−II	-3714.034227	0.06595	-3713.96828

Table S3. Bond lengths (d), energies (*E*) and QTAIM properties of the selected bonds in the investigated systems.

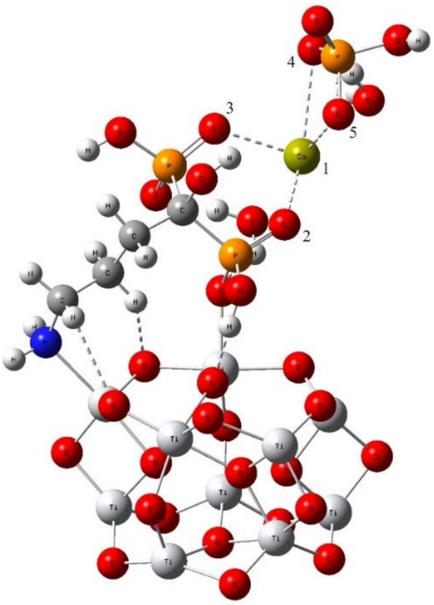

$(TiO_2)_{10}$−alendronate−CaP−I

Bond	d /Å	$\rho(r_c)$/ $e\times a_0^{-3}$	$\nabla^2\rho(r_c)$/ $e\times a_0^{-5}$	$V(r_c)$/au	$G(r_c)$/au	$H(r_c)$/au[a]	E/ kcal mol^{-1} [b]
Ca(1)-O(2)	2.511	2.259×10^{-2}	0.1178	-0.0218	0.0256	0.0038	-6.84
Ca(1)-O(3)	2.517	2.270×10^{-2}	0.1164	-0.0219	0.0255	0.0036	-6.86
Ca(1)-O(4)	2.810	1.302×10^{-2}	0.0599	-0.0115	0.0132	0.0017	-3.61
Ca(1)-O(5)	2.488	2.616×10^{-2}	0.1295	-0.0259	0.0292	0.0032	-8.14

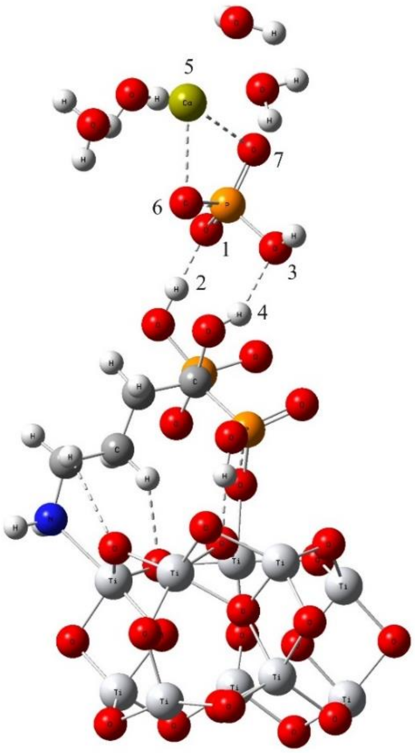

(TiO2)10−alendronate−CaP−II

Bond	d /Å	$\rho(r_c)$/ $e\times a_0^{-3}$	$\nabla^2\rho(r_c)$/ $e\times a_0^{-5}$	$V(r_c)$/au	$G(r_c)$/au	$H(r_c)$/au[a]	E/ kcal mol^{-1}
O(1)-H(2)	1.653	4.709×10^{-2}	0.1483	-0.0367	0.0369	0.0002	-11.51
O(3)-H(4)	1.953	2.539×10^{-2}	0.0742	-0.0201	0.0193	-0.0008	-6.29
Ca(5)-O(6)	2.516	2.465×10^{-2}	0.1216	-0.0242	0.0273	0.0031	-7.61
Ca(5)-O(7)	2.569	2.197×10^{-2}	0.1056	-0.0211	0.0237	0.0026	-6.61

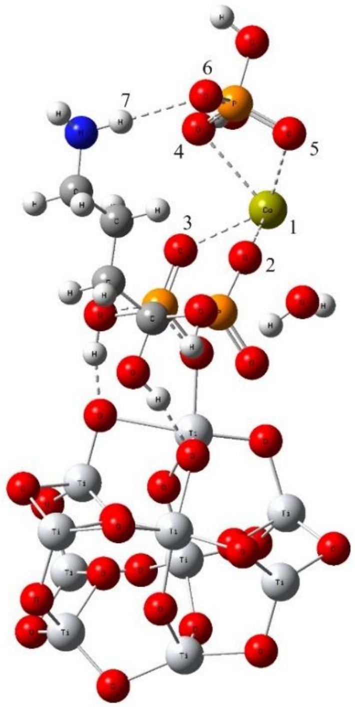

$(TiO_2)_{10}$−alendronate−CaP−III							
Bond	d /Å	$\rho(r_c)$/ $e\times a_0^{-3}$	$\nabla^2\rho(r_c)$/ $e\times a_0^{-5}$	$V(r_c)$/au	$G(r_c)$/au	$H(r_c)$/au	E/ kcal mol^{-1}
Ca(1)-O(2)	2.450	2.594×10^{-2}	0.1406	-0.0259	0.0305	0.0046	-8.11
Ca(1)-O(3)	2.478	2.436×10^{-2}	0.1296	-0.0239	0.0282	0.0043	-7.50
Ca(1)-O(4)	2.663	1.793×10^{-2}	0.0829	-0.0165	0.0186	0.0021	-5.19
Ca(1)-O(5)	2.507	2.511×10^{-2}	0.1244	-0.0248	0.0279	0.0031	-7.79
O(6)-H(7)	2.192	1.678×10^{-2}	0.0454	-0.0127	0.0120	-0.0007	-4.00

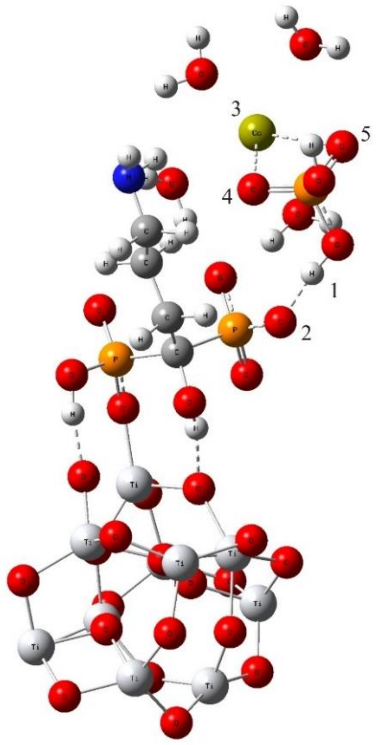

(TiO2)10−alendronate−CaP−IV

Bond	d /Å	$\rho(r_c)$/ $e\times a_0^{-3}$	$\nabla^2\rho(r_c)$/ $e\times a_0^{-5}$	$V(r_c)$/au	$G(r_c)$/au	$H(r_c)$/au	E/ kcal mol^{-1}
H(1)-O(2)	1.533	6.251×10^{-2}	0.1768	-0.0565	0.0504	-0.0061	-17.73
Ca(3)-O(4)	2.559	2.201×10^{-2}	0.1064	-0.0209	0.0238	0.0029	-6.57
Ca(3)-O(5)	2.794	1.308×10^{-2}	0.0615	-0.0114	0.0134	0.0020	-3.59

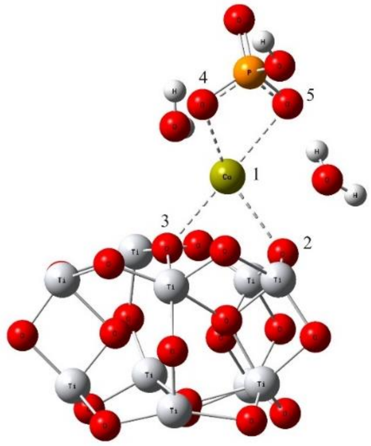

(TiO2)10−CaP−I

Bond	d /Å	$\rho(r_c)$/ $e\times a_0^{-3}$	$\nabla^2\rho(r_c)$/ $e\times a_0^{-5}$	$V(r_c)$/au	$G(r_c)$/au	$H(r_c)$/au	E/ kcal mol^{-1}
Ca(1)-O(2)	2.545	2.258×10^{-2}	0.1118	-0.0220	0.0250	0.0030	-6.89
Ca(1)-O(3)	2.518	2.270×10^{-2}	0.1163	-0.0221	0.0256	0.0035	-6.94
Ca(1)-O(4)	2.506	2.531×10^{-2}	0.1247	-0.0250	0.0281	0.0031	-7.86
Ca(1)-O(5)	2.537	2.378×10^{-2}	0.1155	-0.0233	0.0261	0.0028	-7.31

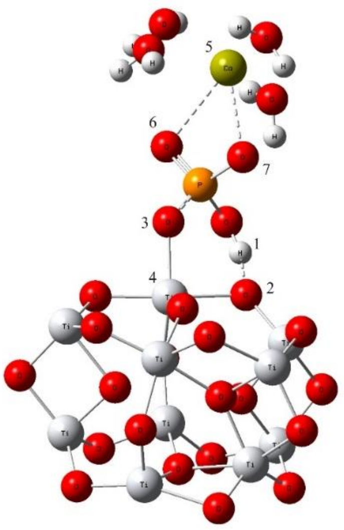

(TiO$_2$)$_{10}$−CaP−II							
Bond	d /Å	$\rho(r_c)$/ $e\times a_0^{-3}$	$\nabla^2\rho(r_c)$/ $e\times a_0^{-5}$	$V(r_c)$/au	$G(r_c)$/au	$H(r_c)$/au	E/ kcal mol^{-1}
H(1)-O(2)	1.727	4.029×10^{-2}	0.1307	-0.0319	0.0323	0.0004	-10.02
O(3)-Ti(4)	1.986	8.553×10^{-2}	0.4677	-0.1148	0.1158	0.0010	-36.00
Ca(5)-O(6)	2.662	1.780×10^{-2}	0.0830	-0.0163	0.0185	0.0022	-5.13
Ca(5)-O(7)	2.524	2.407×10^{-2}	0.1185	-0.0235	0.0265	0.0030	-7.37

$^aH(r_c) = V(r_c) + G(r_c)$; $^bE = 0.5 \times V(r_c)$

Cartesian coordinates of the calculated systems

$(TiO_2)_{10}$

O	0.039305	-0.015425	-0.096029
Ti	-0.142229	-0.016449	1.909261
O	1.643979	-0.081708	2.225577
Ti	2.588875	-1.632763	2.701598
O	1.385534	-2.340195	0.813656
Ti	0.500223	-1.634302	-0.700172
O	-0.835925	-2.909901	-0.425808
Ti	-0.717366	-3.425701	1.376934
O	-0.038901	-3.603219	3.256275
Ti	1.187160	-4.492811	4.427591
O	2.039784	-3.483926	5.822923
Ti	2.122282	-1.690467	5.604155
O	0.976851	-1.661306	4.177928
Ti	-0.446984	-0.773519	4.936094
O	-0.647342	0.552109	3.713348
Ti	-1.574243	-3.883261	4.129682
O	-2.165108	-4.078557	2.176736
O	0.736288	-0.670242	6.371498
O	-1.639573	-2.241342	4.926268
Ti	2.181085	-5.041926	1.774874
O	3.218363	-4.444322	0.285745
Ti	3.032921	-2.661180	-0.012552
O	2.183516	-1.956117	-1.502171
O	0.557956	-4.947496	1.108874
O	-0.982160	-1.540559	1.964775
O	-0.519545	-4.933019	5.194947
O	1.753850	-5.925654	3.534382
O	2.454572	-3.626436	2.916724
O	3.851642	-1.709565	1.363512
O	3.445548	-1.293750	4.274371

$(TiO_2)_{10}$-alendronate-I

O	-0.130932	-6.339375	1.069645
O	0.945192	-7.380542	0.758953
O	1.844097	-7.777265	1.916812
O	2.797740	-8.896973	1.550925
N	3.383172	-9.492688	2.774376
P	0.533056	-4.672737	1.588314
O	1.835091	-4.466089	0.611637

O	0.974999	-4.695431	3.033674
O	-0.479580	-3.650592	1.114407
P	-1.422684	-6.936676	2.276451
O	-0.801859	-7.273441	3.658081
O	-2.557362	-5.944618	2.294690
O	-1.918649	-8.325224	1.611710
O	-0.837104	-6.142427	-0.161415
H	1.537250	-6.993835	-0.083547
H	0.429257	-8.279360	0.385585
H	2.423812	-6.918619	2.289691
H	1.226020	-8.115583	2.752103
H	2.263276	-9.696574	1.024089
H	3.591156	-8.534259	0.882022
H	-1.315417	-5.298376	-0.094011
H	3.883812	-8.766521	3.289501
H	4.083724	-10.183855	2.506820
H	-1.630351	-9.104515	2.155913
H	2.626294	-4.911963	0.950536
Ti	-3.010550	-13.691269	5.070751
Ti	-0.946458	-11.484075	6.866577
Ti	-3.866488	-11.720766	7.216836
Ti	-0.849381	-8.981525	4.761221
Ti	1.871866	-10.498133	4.149169
Ti	1.713321	-12.464791	6.172058
Ti	-3.880534	-10.035909	4.943603
Ti	-0.418958	-12.063234	2.919268
Ti	-3.316033	-12.281534	2.596649
Ti	-0.264748	-14.540617	5.074188
O	1.413641	-14.272074	5.875670
O	1.252681	-11.375126	2.566215
O	-4.188042	-13.586592	3.534917
O	-4.963447	-10.415746	6.421487
O	-0.780950	-9.715032	6.514883
O	-4.114811	-13.420241	6.608158
O	-1.557669	-12.134290	1.538335
O	-2.179629	-15.245948	4.830461
O	-2.828200	-8.636780	5.076927
O	2.998640	-11.366907	5.335550
O	-2.234229	-11.636711	8.184449
O	-2.086081	-12.458372	3.881541
O	-1.221370	-13.372824	6.108383
O	-1.037251	-10.080029	3.450358
O	0.516609	-11.677156	5.050400
O	-2.796836	-11.320528	5.777003
O	-4.062512	-10.729707	3.179662
O	0.034492	-13.902454	3.442468

O	1.190331	-9.000501	4.787222
O	0.722266	-11.926225	7.627469

$(TiO_2)_{10}$-alendronate-II

C	-0.497480	-8.041580	9.762053
C	-1.573665	-7.394967	10.641207
C	-1.223045	-6.157728	11.453235
C	-2.448877	-5.634422	12.176015
N	-2.105191	-4.499040	13.032760
P	0.979471	-8.659216	10.725135
O	0.259159	-9.492427	11.945516
O	1.723838	-7.523784	11.390367
O	1.737824	-9.629895	9.843011
P	0.018485	-6.909691	8.377461
O	0.821789	-5.730091	8.848137
O	0.732416	-7.795364	7.312349
O	-1.364403	-6.417581	7.726540
O	-1.153674	-9.173594	9.187630
H	-1.930558	-8.183440	11.319711
H	-2.419516	-7.161538	9.977137
H	-0.444171	-6.392302	12.193692
H	-0.815776	-5.366079	10.808577
H	-3.193349	-5.301357	11.440494
H	-2.914897	-6.464724	12.735554
H	-0.562551	-9.651970	8.565844
H	-1.455549	-4.820631	13.748133
H	-2.936256	-4.189187	13.530199
H	-1.677980	-7.100041	7.057250
H	-0.090106	-10.344063	11.643007
Ti	-3.509721	-12.798200	3.493948
Ti	-0.607317	-12.398665	6.754625
Ti	-3.443836	-11.784568	6.601320
Ti	0.375249	-9.255684	6.037605
Ti	2.348115	-10.941256	4.349560
Ti	1.818380	-13.498197	5.360478
Ti	-2.499042	-9.565048	5.052520
Ti	-0.261265	-10.931590	2.930720
Ti	-3.062873	-10.315740	2.194190
Ti	-0.751514	-14.149351	3.729481
O	1.094559	-14.678231	4.216605
O	1.562457	-10.652599	2.690657
O	-4.252149	-11.796664	2.125951
O	-4.023257	-10.138341	6.167603

O	0.184016	-10.595980	7.169150
O	-4.163672	-12.803691	5.247206
O	-1.280241	-10.205230	1.574938
O	-2.586203	-14.265472	3.020650
O	-1.804519	-8.325326	6.036640
O	3.302219	-12.448434	4.797938
O	-1.992055	-12.666198	7.822255
O	-2.322390	-11.334200	3.553961
O	-1.213218	-13.695656	5.335437
O	-0.498674	-9.631946	4.349471
O	0.889421	-11.929363	4.888413
O	-1.904001	-11.262640	5.839792
O	-3.345055	-9.001245	3.391815
O	-0.298678	-12.781322	2.632061
O	2.119148	-9.467234	5.455164
O	0.954507	-13.458364	6.995759

$(TiO_2)_{10}$-alendronate-CaP-I

C	0.009146	0.628388	0.300223
O	0.014991	0.577697	1.730176
C	1.483249	0.624986	-0.110016
C	1.734699	0.717156	-1.603634
C	3.202460	0.802465	-1.963789
N	3.350248	1.163302	-3.393917
Ti	2.447975	3.175765	-3.972256
O	0.764228	2.651830	-3.901929
Ti	-0.425794	3.940548	-2.860697
O	0.814641	4.581183	-1.855066
Ti	2.727015	5.526100	-2.072851
O	3.940333	6.702065	-3.076368
Ti	3.395001	7.720991	-4.426827
O	1.574949	7.702918	-4.615247
Ti	0.135832	6.304356	-5.052030
O	1.031163	5.725827	-6.608588
Ti	2.663607	5.166832	-5.965877
O	1.992200	5.109485	-4.275746
P	-0.936291	-0.869236	-0.296149
O	-2.145639	-1.000644	0.611109
P	-0.889029	2.209212	-0.128197
O	0.079136	3.367371	0.431819
O	0.049327	-2.108032	0.089148
O	-1.163574	-0.826721	-1.794811
O	-1.023556	2.386530	-1.664366

O	-2.190251	2.225036	0.639784
Ti	-1.582447	8.428244	-3.937697
O	-0.524768	7.132114	-3.176888
Ti	-1.593879	6.700110	-1.697173
O	-0.395560	6.940051	-0.236884
Ti	1.334938	7.474191	-0.396200
O	1.547970	7.097013	-2.130374
Ti	1.239767	8.857171	-2.902209
O	2.855975	9.414425	-3.394687
O	1.225599	9.198737	-0.994498
O	-0.323625	9.703398	-3.606607
O	-0.753279	4.820578	-4.514929
O	-1.151944	7.531625	-5.555822
O	-2.690987	7.998093	-2.479929
O	-1.958098	5.025649	-2.080189
O	3.423431	3.870554	-2.481009
O	3.100310	3.351807	-5.697251
O	3.812215	6.622663	-5.893371
O	2.775436	6.005952	-0.347730
H	1.939616	-0.281238	0.315063
H	1.964190	1.478262	0.392263
H	1.281534	-0.134400	-2.132997
H	1.240486	1.610991	-1.991525
H	3.697845	1.583484	-1.375025
H	3.716090	-0.146363	-1.752814
H	-0.897230	0.458339	2.042251
H	2.923049	0.431545	-3.964411
H	4.340258	1.167218	-3.638152
H	0.484104	3.892122	-0.310326
H	0.735474	-2.257212	-0.579865
Ca	-4.089254	0.592409	0.463017
P	-4.641116	-0.448796	3.450364
O	-3.891890	-1.147552	4.581524
O	-5.037282	-1.348242	2.261377
O	-6.127208	0.005252	4.050352
O	-3.964997	0.839431	2.935919
O	-6.787294	-0.046651	0.662809
H	-6.363529	-0.666509	1.302102
H	-7.144512	-0.601626	-0.040215
O	-3.448604	0.795068	-2.039938
H	-2.938274	1.619830	-2.090920
H	-2.739046	0.118020	-2.073255
H	-6.034764	0.532703	4.855850

$(TiO_2)_{10}$-alendronate-CaP-II

C	0.036678	-0.278413	-0.225590
O	0.104072	-0.450452	1.190945
C	1.486118	-0.456324	-0.699389
C	1.770234	-0.072454	-2.138352
C	3.243182	-0.035808	-2.483375
N	3.420105	0.452613	-3.873734
Ti	2.530804	2.486184	-4.316741
O	0.847065	1.962248	-4.246374
Ti	-0.343006	3.250979	-3.205100
O	0.897468	3.891615	-2.199507
Ti	2.809852	4.836527	-2.417312
O	4.023162	6.012492	-3.420821
Ti	3.477837	7.031415	-4.771277
O	1.657783	7.013342	-4.959697
Ti	0.218682	5.614769	-5.396488
O	1.113997	5.036252	-6.953037
Ti	2.746452	4.477268	-6.310314
O	2.075027	4.419907	-4.620195
P	-1.131132	-1.562400	-0.926287
O	-2.486027	-1.363403	-0.267883
P	-0.632379	1.444829	-0.487101
O	0.599962	2.390468	-0.031946
O	-0.475443	-2.946386	-0.387731
O	-1.051084	-1.569357	-2.438502
O	-0.902112	1.692836	-2.000306
O	-1.808874	1.676440	0.428725
Ti	-1.499613	7.738668	-4.282147
O	-0.441934	6.442538	-3.521338
Ti	-1.511044	6.010533	-2.041619
O	-0.312726	6.250475	-0.581334
Ti	1.417772	6.784611	-0.740645
O	1.630799	6.407433	-2.474819
Ti	1.322601	8.167595	-3.246659
O	2.938809	8.724849	-3.739137
O	1.308433	8.509161	-1.338948
O	-0.240791	9.013822	-3.951057
O	-0.670423	4.130986	-4.859392
O	-1.069110	6.842049	-5.900272
O	-2.608153	7.308517	-2.824379
O	-1.875264	4.336081	-2.424649
O	3.506278	3.180983	-2.825470
O	3.183145	2.662234	-6.041700
O	3.895051	5.933085	-6.237823
O	2.858271	5.316376	-0.692180

H	1.774558	-1.499408	-0.501438
H	2.104739	0.165237	-0.033775
H	1.252782	-0.749471	-2.832440
H	1.360264	0.920379	-2.332590
H	3.777756	0.655021	-1.818840
H	3.704261	-1.027364	-2.366592
H	-0.779245	-0.611716	1.576624
H	2.990284	-0.218303	-4.513095
H	4.413354	0.462414	-4.104174
H	0.861484	3.004822	-0.769779
H	-0.833319	-3.213151	0.510051
P	-1.146707	-2.977661	3.233207
O	0.358659	-2.756380	3.474334
O	-1.478165	-3.732597	1.940957
O	-1.784034	-3.559871	4.506751
O	-1.836895	-1.487073	2.965542
Ca	0.468492	-3.991774	5.663762
O	0.335672	-6.179151	4.358924
H	0.764169	-6.030392	3.505045
H	-0.599114	-6.298718	4.141902
O	-0.058767	-2.171890	7.348906
H	-0.983445	-2.153585	7.627885
H	0.122106	-1.275548	7.037584
O	-1.137958	-5.219486	7.217640
H	-1.917955	-4.653196	7.291004
H	-1.464255	-6.029249	6.803521
O	2.883445	-4.237655	4.844477
H	3.298478	-5.101615	4.960757
H	2.872246	-4.095327	3.888619
H	-1.753404	-0.917538	3.744065

$(TiO_2)_{10}$-alendronate-CaP-III

C	0.412074	0.156708	-0.360987
O	0.511488	0.096619	1.060707
C	1.847942	-0.044515	-0.859603
C	2.092948	-0.015150	-2.360932
C	3.486983	-0.498520	-2.704408
N	3.748797	-0.338407	-4.135373
P	-0.748333	-1.206112	-0.912111
O	-1.930516	-1.239808	0.029207
P	-0.240837	1.832405	-0.823033
O	0.832828	2.867029	-0.252918
O	0.161380	-2.550225	-0.701005

O	-1.006157	-1.132522	-2.405666
O	-0.397276	2.011975	-2.311538
O	-1.571678	2.030112	-0.030418
Ti	-2.177861	2.278957	1.856031
O	-3.990029	2.149386	1.503283
Ti	-5.034128	2.114848	3.038273
O	-3.762727	1.522445	4.232888
Ti	-3.446497	3.920449	4.782224
O	-3.977515	3.114591	6.388685
Ti	-3.399349	1.603816	7.203655
O	-2.160409	0.731692	6.365483
Ti	-1.728889	0.199375	4.469745
O	-3.201374	-1.004727	4.517978
Ti	-4.703454	0.017948	4.864243
O	-5.993119	0.657094	3.619860
Ti	0.774646	1.421351	5.297723
O	-0.776055	1.901496	4.530772
Ti	-0.237070	3.635958	3.784564
O	-0.145468	5.475360	4.416833
Ti	-1.240790	5.588826	5.841341
O	-1.484931	3.754355	5.743041
Ti	-0.831179	3.286889	7.449129
O	-2.178579	2.578464	8.404342
O	-0.593065	5.118699	7.565167
O	0.534711	2.090739	6.996177
O	-1.802013	0.687765	2.519401
O	-0.111887	-0.416457	4.837118
O	1.451024	2.846887	4.435018
O	-0.094745	3.386838	2.080052
O	-2.291333	3.614217	3.255786
O	-5.056762	3.780805	3.860838
O	-4.880331	0.433281	6.602361
O	-3.028885	5.660262	5.232451
H	2.179172	-1.011404	-0.453512
H	2.467073	0.722099	-0.370315
H	1.361143	-0.650268	-2.881647
H	1.954252	1.002004	-2.754478
H	4.230499	0.082879	-2.139932
H	3.587463	-1.544455	-2.363274
H	-0.364297	0.241307	1.483155
H	3.000246	-0.794320	-4.664310
H	4.610385	-0.819917	-4.378519
H	0.592975	3.134430	0.692261
H	0.211045	-2.814667	0.230052
Ca	-1.818578	0.678513	-3.842078
P	0.176545	-0.429218	-6.000222

O	0.448205	0.882016	-5.224557
O	1.296321	-1.460382	-5.871235
O	-1.240699	-0.931455	-5.674477
O	0.069108	-0.020267	-7.607501
O	-0.996118	3.052597	-6.027858
H	-0.631154	3.827122	-5.584616
H	-0.401230	2.306683	-5.774988
O	-3.515160	1.116725	-1.968709
H	-3.023677	1.481094	-1.208644
H	-4.262754	1.712844	-2.097005
H	0.943175	0.172036	-7.974918

$(TiO_2)_{10}$-alendronate-CaP-IV

C	-0.334307	-0.187518	-0.165730
O	-0.221034	-0.163958	1.263330
C	1.116448	-0.298166	-0.649130
C	1.387286	-0.165548	-2.138857
C	2.828540	-0.495737	-2.470571
N	3.054675	-0.429159	-3.916311
P	-1.385274	-1.682600	-0.635864
O	-2.677649	-1.557605	0.179928
P	-1.091109	1.427232	-0.688740
O	-0.002043	2.537182	-0.265122
O	-0.533366	-2.908902	-0.215511
O	-1.632634	-1.641566	-2.164481
O	-1.366841	1.523074	-2.165226
O	-2.344475	1.677563	0.207965
Ti	-2.859111	1.947670	2.088856
O	-4.686885	1.878049	1.806777
Ti	-5.673259	1.941214	3.378593
O	-4.381840	1.342199	4.548178
Ti	-3.952692	3.744004	4.994679
O	-4.453894	3.019025	6.648555
Ti	-3.904470	1.516945	7.498720
O	-2.732496	0.565985	6.650041
Ti	-2.393269	-0.051787	4.761476
O	-3.908396	-1.193388	4.908231
Ti	-5.355744	-0.099448	5.269462
O	-6.665457	0.545163	4.049068
Ti	0.184951	1.098229	5.451915
O	-1.373672	1.611892	4.723719
Ti	-0.796335	3.295083	3.893514
O	-0.609993	5.151118	4.452054

Ti	-1.645930	5.360104	5.909788
O	-1.964381	3.534524	5.889797
Ti	-1.265706	3.103607	7.587683
O	-2.602749	2.484935	8.616927
O	-0.952711	4.927416	7.625705
O	0.034938	1.838022	7.131378
O	-2.520507	0.367881	2.797958
O	-0.788671	-0.718128	5.093383
O	0.883162	2.463206	4.512106
O	-0.727756	2.978633	2.195623
O	-2.868279	3.336430	3.440289
O	-5.600563	3.635555	4.137845
O	-5.451134	0.385579	6.995872
O	-3.451491	5.480915	5.363447
H	1.485176	-1.275505	-0.301040
H	1.698604	0.459824	-0.102954
H	0.733641	-0.849052	-2.694774
H	1.163488	0.851990	-2.490840
H	3.496867	0.226138	-1.981417
H	3.075909	-1.485863	-2.046682
H	-1.104063	-0.091401	1.683236
H	2.505718	-1.164235	-4.361958
H	4.026996	-0.649971	-4.116644
H	-0.135698	2.780351	0.698376
H	0.065616	-4.063100	-1.026458
P	1.124035	-4.697121	-2.876436
O	0.585350	-5.691241	-3.889526
O	0.452308	-4.910258	-1.448233
O	2.668893	-5.059871	-2.502159
O	1.000459	-3.253590	-3.330920
Ca	-0.616510	-3.590268	-5.286131
O	-0.219416	-5.408872	-7.055222
H	0.489204	-5.171047	-7.666965
H	0.096577	-6.207999	-6.613471
O	-0.988087	-1.129292	-4.742797
H	-1.161282	-1.199235	-3.772904
H	-0.187118	-0.595103	-4.818887
O	-2.330776	-3.931636	-3.428080
H	-2.114175	-3.127666	-2.888341
H	-2.055992	-4.680951	-2.884077
O	1.472194	-2.783319	-6.529829
H	1.631249	-1.835295	-6.431332
H	1.484783	-2.939496	-7.482843
H	3.218264	-5.159491	-3.293757

$(TiO_2)_{10}$-CaP-I

Ti	-2.412379	-12.961136	4.921309
Ti	-0.483525	-10.588818	6.706684
Ti	-3.413056	-10.791370	6.829102
Ti	-0.124207	-8.267055	4.387492
Ti	2.611606	-9.809145	4.116842
Ti	2.235683	-11.627220	6.267847
Ti	-3.216227	-9.211741	4.502801
Ti	0.368685	-11.460140	2.867277
Ti	-2.512138	-11.685808	2.359614
Ti	0.319933	-13.792578	5.212879
O	1.948993	-13.442912	6.088226
O	2.083299	-10.900290	2.595146
O	-3.464171	-12.938155	3.271151
O	-4.409910	-9.488182	5.941061
O	-0.277999	-8.877858	6.210125
O	-3.665891	-12.525864	6.304867
O	-0.689592	-11.654141	1.426366
O	-1.587280	-14.526098	4.895120
O	-2.139974	-7.816521	4.505941
O	3.598922	-10.557252	5.453388
O	-1.880191	-10.633148	7.920613
O	-1.405097	-11.790217	3.733302
O	-0.709406	-12.528169	6.044454
O	-0.267074	-9.491123	3.209493
O	1.175477	-10.942061	4.977330
O	-2.225723	-10.460745	5.457853
O	-3.324239	-10.079063	2.820781
O	0.718260	-13.290880	3.572941
O	1.884003	-8.470350	5.131195
O	1.134198	-10.991382	7.592023
Ca	-1.833607	-8.482329	1.515309
P	-1.820882	-7.693548	-1.467151
O	-1.755292	-6.322952	-2.132986
O	-1.939893	-8.827201	-2.671642
O	-0.585160	-8.054530	-0.615435
O	-3.091578	-7.965881	-0.626026
O	-1.214825	-6.055801	1.860619
H	-1.803239	-5.489310	2.375940
H	-1.099457	-5.597716	1.017118
O	-3.430359	-10.713787	-0.334506
H	-3.400294	-9.742859	-0.495972
H	-4.305615	-10.877950	0.036797
H	-1.294952	-8.663972	-3.373966

$(TiO_2)_{10}$-CaP-II

Ti	-3.732695	-10.027525	2.829264
Ti	-1.802259	-10.548809	5.751368
Ti	-3.487146	-8.190788	5.261302
Ti	1.128700	-9.433446	4.717673
Ti	1.493393	12.539605	4.312689
Ti	-0.999258	-13.358963	5.403256
Ti	-1.183262	-7.331843	3.883389
Ti	-0.301818	-11.415707	2.114857
Ti	-1.698185	-9.059645	1.062259
Ti	-3.095921	-12.799368	3.221129
O	-2.404919	-14.062262	4.433530
O	1.099219	-12.545760	2.407599
O	-3.510981	-8.992018	1.183523
O	-2.632261	-6.586961	4.839871
O	-0.213254	-9.777272	6.060413
O	-4.662067	-8.851480	4.024222
O	-0.337229	-10.506396	0.563668
O	-4.459796	-11.550022	2.296244
O	0.390084	-7.503343	4.658358
O	0.843624	-13.878627	5.364338
O	-3.082633	-9.491286	6.569359
O	-1.828696	-10.162390	2.437106
O	-3.053359	-11.329152	4.310306
O	0.678125	-9.865548	3.131820
O	-0.453136	-11.995659	4.353648
O	-2.030970	-8.907026	4.386061
O	-1.127664	-7.561753	2.002596
O	-1.792353	-12.736606	2.039393
O	1.938824	-11.089944	5.291456
O	-1.677075	-12.200530	6.656044
H	1.477965	-6.734766	5.757168
O	2.933532	-8.605306	4.741104
P	3.493673	-7.206459	5.171839
O	3.676742	-6.282656	3.961985
O	2.365168	-6.570398	6.163907
O	4.770414	-7.298598	6.001216
Ca	5.961307	-5.397367	4.567637
O	6.215586	-5.071410	2.045766
H	6.724739	-5.789668	1.648093
H	5.348419	-5.124225	1.622908
O	7.190469	-7.511393	3.812461
H	7.006511	-8.186027	4.479949
H	6.820173	-7.868898	2.994887
O	4.809831	-3.956005	6.336500

H	3.901938	-4.265245	6.460040
H	5.230817	-4.088296	7.196089
O	7.705627	-5.802617	6.392577
H	7.529399	-6.677625	6.763803
H	7.643027	-5.200590	7.145138
